# The BH3-only protein BAD mediates TNFα cytotoxicity despite concurrent activation of IKK and NF-κB in septic shock

**DOI:** 10.1038/s41422-018-0041-7

**Published:** 2018-05-24

**Authors:** Jie Yan, Hao Zhang, Jialing Xiang, Yu Zhao, Xiang Yuan, Beicheng Sun, Anning Lin

**Affiliations:** 1 Ben May Department for Cancer Research, The University of Chicago, Chicago, IL 60637 USA; 2 The Second Affiliated Hospital, The State Key Laboratory of Respiratory Disease, Guangdong Provincial Key Laboratory of Allery & Clinical Immunology, Guangzhou Medical University, Guangzhou, Guangdong, 510260 China; 3 The State Key Laboratory of Cell Biology, CAS Center for Excellence in Molecular Cell Science, Shanghai Institute of Biochemistry and Cell Biology, Chinese Academy of Sciences, University of Chinese Academy of Sciences, Shanghai, 200031 China; 4 Department of Biology, Illinois Institute of Technology, Chicago, IL 60616 USA; 5 Department of Hepatobiliary Surgery, The Affiliated Drum Tower Hospital of Nanjing University Medical School, Nanjing, China

## Abstract

The inflammatory cytokine TNFα plays a crucial role in the pathology of many inflammatory and infectious diseases. However, the mechanism underlying TNFα cytotoxicity in these diseases is incompletely understood. Here we report that the pro-apoptotic BCL-2 family member BAD mediates TNFα cytotoxicity despite concurrent activation of IKK and NF-κB in vitro by inducing apoptosis in cultured cells and in vivo by eliciting tissue damage of multiple organs and contributing to mortality in septic shock. At high doses, TNFα significantly inactivates RhoA through activation of the Src-p190GAP pathway, resulting in massive actin stress fiber destabilization, followed by substantial BAD release from the cytoskeleton to the cytosol. Under this condition, activated IKK fails to phosphorylate all cytosolic BAD, allowing translocation of non-phosphorylated BAD to mitochondria to trigger apoptosis. Polymicrobial infection utilizes the same mechanism as high-dose TNFα to elicit apoptosis-associated tissue damage of multiple organs. Consequently, loss of *Bad* or elimination of BAD pro-apoptotic activity protects mice from tissue damage of multiple organs and reduces mortality rates. Our results support a model in which BAD mediates TNFα cytotoxicity despite concurrent activation of the IKK-NF-κB pathway in cultured mammalian cells and in septic shock.

## Introduction

The inflammatory cytokine TNFα plays a crucial role in inflammatory and infectious diseases, from rheumatoid arthritis to septic shock.^1^ The anti-TNFα therapy has been limited to the treatment of certain inflammatory diseases such as rheumatic arthritis, due to the side effects caused by inevitably abrogating TNFα-mediated beneficial inflammatory response in the host defense against pathogens.^2–4^ Furthermore, the molecular mechanism underlying TNFα cytotoxicity in disease is obscure, hindering the development of more efficient anti-TNFα-based therapies.

Overwhelming evidence shows that TNFα does not typically induce apoptosis unless the IκB kinase (IKK) signaling pathway is impaired in cultured cells and various genetic animal models. TNFα-induced apoptosis in most cell types is suppressed by IKK, which has two catalytic subunits, IKKα and IKKβ, and two regulatory subunits NEMO/IKKγ and ELKS.^5^ IKK inhibits TNFα-induced apoptosis through activation of the transcription factor NF-κB, whose target gene protein products include inhibitors of caspases (IAPs),^6–8^ and prevents prolonged activation of JNK1.^9–13^ In addition to activation of NF-κB, IKK-mediated phosphorylation and inactivation of the BH3-only protein BAD is also required for suppressing TNFα-induced apoptosis.^14^

Sepsis is a life-threatening disease with organ dysfunction caused by a dysregulated host response to infection and septic shock is a subset of sepsis in which underlying circulatory and cellular metabolism abnormalities are profound enough to substantially increase mortality.^15–17^ Although the pathology of sepsis and septic shock has yet to be fully understood, it is generally recognized that an over-reactive immune response, excessive production of cytokines including TNFα and IL-1β, microcirculatory malfunction such as vasodilatation and coagulopathy, and cardiovascular shock could lead to multiple organ damage and ultimate mortality.^2–4, 15–17^ The molecular mechanism underlying multiple organ damage is incompletely understood.

Although the role of apoptosis in multiple organ damage during septic shock is controversy, having either promoting or inhibiting effect in a cell type-dependent manner,^18–24^ it has been generally recognized that TNFα-induced apoptosis is a crucial promoting factor. Aberrant elevation of TNFα production is known to induce while genetic disruption of its receptor (TNF-R1) reduces tissue damage of multiple organs and mortality in septic shock.^25–28^ Furthermore, overly production of TNFα is responsible for the activation of secondary inflammatory response that produces pro- and anti-inflammatory cytokines, lipids mediators, reactive oxygen species (ROS) and cell adhesion molecules in lipopolysaccharide (LPS; also known as endotoxin)-induced septic shock.^29^ TNFα also induces production of nitric oxide, which is known to cause microcirculatory malfunction in septic shock.^30^ In patients, a polymorphism in the TNF promoter results in overly higher TNFα levels and worse prognosis.^27^ Thus, TNFα cytotoxicity is likely to be a major factor contributing to the pathogenesis of septic shock. However, the signaling mechanism underlying TNFα cytotoxicity in septic shock is not known.

The pro-apoptotic BCL-2 family member BAD is required for the activation of mitochondrial death machinery by a variety of death stimuli such as withdrawal of growth factors and hematopoietic cytokines^31–33^ and TNFα.^31–34^ Like all other BH3-only proteins, BAD is a potent inducer of apoptosis even though it does not directly induce apoptosis.^32, 35^ The pro-apoptotic activity of BAD is suppressed by the phosphorylation-mediated sequestration in the cytosol. In response to growth and survival factors, BAD is phosphorylated at Ser112, Ser136, and Ser155 (the regulatory “serines”) by several protein kinases including PKA, Raf-1, Akt/PKB, Rsk2, and CaMKII or at Thr201 by JNK1^32, 33, 36–46^ and further sequestered in the cytosol through interaction with the cytosolic anchorage protein 14-3-3.^47^ Phosphorylated BAD is an important regulator of glucose metabolism that contributes to cell survival.^31–33^ Upon withdrawal of growth and survival factors, non-phosphorylated BAD dissociates from 14-3-3 and subsequently translocates to mitochondrial membrane, where it binds to and replaces the anti-apoptotic BCL-2 family member BCL-2 from BCL-2:BAK and BCL-X_L_ from BCL-X_L_:BAK heterodimers, respectively, thereby promoting the pro-apoptotic BCl-2 family member BAK to form oligomers with another pro-apoptotic BCL-2 family member BAX to induce apoptosis.^32–35^ In response to TNFα, BAD is phosphorylated at Ser26 by IKKβ and the phosphorylation is a pre-requisite for BAD to be further phosphorylated at the “regulatory serines” by other protein kinases, thereby preventing BAD from translocation to mitochondria to induce apoptosis.^14^ Elimination of IKK-mediated phosphorylation of BAD accelerates TNFα-induced apoptosis when NF-κB activation is impaired in cultured cells and in animals.^14^ Thus, BAD is required for TNFα-induced apoptosis when IKK activation is impaired.^14^ However, the pathological role of BAD in disease is poorly understood. It is also puzzling that aberrant elevation of TNFα production elicits tissue damage in many inflammatory and infectious diseases including septic shock,^1, 48^ in which there are few known genetic defects in or pharmaceutical inhibitors of the IKK signaling pathway. Here we report that BAD is essential for mediating TNFα cytotoxicity for tissue damage of multiple orgrans and mortality despite concurrent activation of the IKK-NF-κB pathway in septic shock.

## Results

### High-dose TNFα is sufficient to induce BAD-dependent apoptosis despite concurrent activation of IKK and NF-κB

**Fig. 1:**
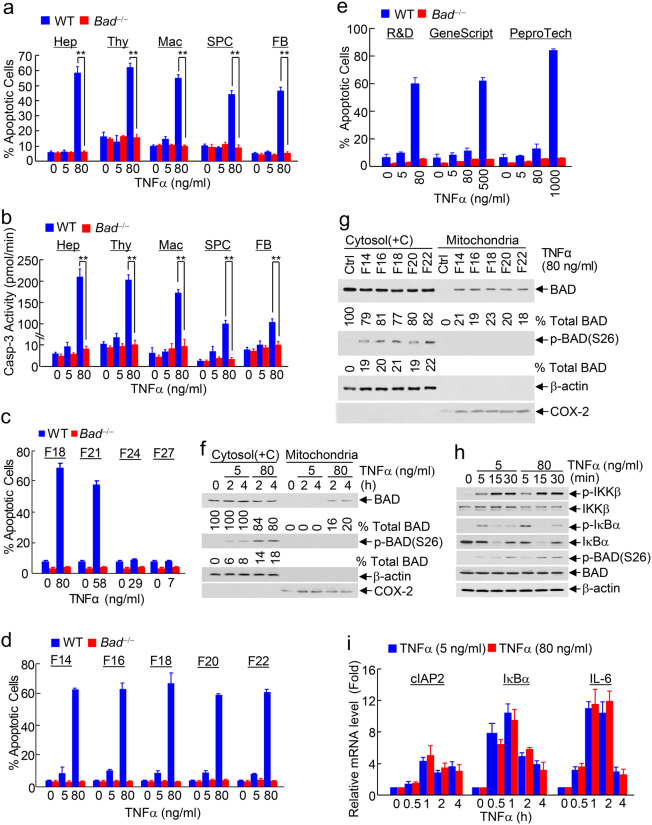
Cytotoxic dose TNFα is sufficient to induce apoptosis in a BAD-dependent manner despite concurrent activation of IKK and NF-κB. **a**, **b** Various primary wild-type (WT) and *Bad*^−/−^ cells were treated without or with non-cytotoxic (5 ng/ml) or cytotoxic (80 ng/ml) dose TNFα for 12 h, as indicated. Apoptotic cells were detected by Annexin V/Propidium iodide (PI) staining and analyzed by flow cytometric analysis (**a**) or determined by caspase-3 activity assay (**b**). Hep hepatocyte, Thy thymocyte, Mac macrophage, SPC splenocyte, FB fibroblast. **c**, **d** WT and *Bad*^−/−^ embryonic fibroblasts were treated without or with various doses of different fractions (F, fraction; F18, fraction number 18 and etc.) of the TNFα preparation (R&D) (TNFα hereinafter for the simplicity unless otherwise specified) for 12 h. Apoptotic cells were determined as described in (**a**). **e** WT and *Bad*^−/−^ embryonic fibroblasts were treated without or with various doses of TNFα from three different commercial sources (R&D, GeneScript and PeproTech; Supplementary information, Table S4) for 24 h. Apoptotic cells were determined as described in **a**. 80 ng/ml TNFα from R&D, 500 ng/ml TNFα from GeneScript, 1000 ng/ml TNFα from PeproTech have similar biological activity (Supplementary information, Table S4). **f**, **g** Cytotoxic but not non-cytotoxic dose TNFα from the pooled TNFα preparation (**f**) or cytotoxic dose TNFα from different fractions of the TNFα preparation (**g**) induced BAD mitochondrial translocation in primary WT hepatocytes (**f**) and WT fibroblasts (**g**), as determined by the cytosol [containing cytoskeleton, Cytosol (+C)] and mitochondria fractionation (see “Materials and methods” for details), followed by immunoblotting with corresponding antibodies. β-Actin and COX-2 were used as the marker of the cytosol and mitochondria, respectively. The percentage of IKK-phosphorylated BAD in total cytoplasmic BAD protein was determined (Supplementary information, Figure S9a-S9b), as described in “Materials and methods”. **h** Cytotoxic and non-cytotoxic dose TNFα induced comparable activation of IKK and NF-κB in primary hepatocytes. Phosphorylation of IKKβ, IκBα and BAD, as well as their protein levels were analyzed by immunoblotting with corresponding antibodies. **i** Primary WT and *Bad*^−/−^ hepatocytes were treated without or with non-cytotoxic or cytotoxic dose TNFα for various durations, as indicated. Total RNA was extracted for quantitative real-time PCR analysis with different primers specifically for cIAP2, IκBα, and IL-6

To determine whether TNFα by itself is able to induce apoptosis, we tested different doses of TNFα in immortalized embryonic fibroblasts (MEFs). Interestingly, TNFα by itself induced apoptosis in MEFs in a dose- and time-dependent manner, as measured by apoptotic cell death assay with Annexin V/Propidium iodide (PI) staining (Supplementary information, Figure S1a, left panel, and S1b). A pulse-stimulation by high-dose TNFα (80 ng/ml, 30 min) was also sufficient to induce apoptosis in wild-type (WT) MEFs (Supplementary information, Figure S1a, right panel), suggesting that rapid change in certain cellular activity is sufficient to mediate the apoptotic activity of high-dose TNFα. At high dose (80 ng/ml; cytotoxic dose hereinafter), but not low dose (5 ng/ml; non-cytotoxic dose hereinafter), TNFα by itself significantly induced apoptosis in WT but not *Bad*^−/−^ primary hepatocytes, thymocytes, macrophages, splenocytes, and fibroblasts, as measured by apoptotic cell death assay (Fig. 1a) or by caspase 3 (Casp-3) activity assay (Fig. 1b). In addition, cytotoxic dose TNFα induced apoptosis in other cell types including HeLa cells in a BAD-dependent manner (Supplementary information, Figure S1c) and Jurkat cells (Supplementary information, Figure S1d). It is interesting to note that Jurkat cells were more sensitive to TNFα than other cell types examined, suggesting that the ability of TNFα to induce apoptosis may vary in different cell types. The apoptotic activity of cytotoxic dose TNFα was blocked in *Tnf*-R1^−/−^ fibroblasts (Supplementary information, Figure S1e), demonstrating that the apoptosis is mediated by TNF-R1 signaling, rather than some unknown off-target effects. Under the same conditions, however, cytotoxic dose TNFα did not induce necroptosis, as analyzed by the MLKL trimerization, which is one of the hall markers of necroptosis (Supplementary information, Figure S1f, Left panel). This is not the result of the disruption of the necroptotic cell death by Bad-deficiency, since non-cytotoxic dose TNFα was able to induce necroptotic cell death (Supplementary information, Figure S1f, Right panel), as previously reported,^49, 50^ in the presence of cycloheximide (CHX) and z-VAD in *Bad*^−/−^ MEFs. Thus, cytotoxic dose TNFα by itself is sufficient to induce apoptosis in a BAD-dependent manner.

It is possible that the apoptosis induced by cytotoxic dose TNFα without blocking the IKK signaling pathway could be due to the sensitization of TNFα cytotoxicity by some unknown pro-apoptotic agent(s) contaminated in the TNFα preparation (R&D; TNFα for the simplicity hereinafter unless specified elsewhere). To exclude this possibility, we first determined the purity of TNFα preparation. MS/MS mass spectrometry revealed that the TNFα preparation contained only TNFα with no detectable chemistry modifications (Supplementary information, Table S1) and had no any chemicals with the molecular weight between 100–2000 Daltons (Supplementary information, Table S2). To definitively exclude the contamination possibility, we used different fractions of the TNFα preparation obtained from ionic exchange chromatograph (Supplementary information, Figure S1g and Table S3), since under this condition the putative contaminant(s) cannot co-elute with TNFα in various chromatographic fractions. Indeed, different fractions of the TNFα preparation induced apoptosis in WT but not *Bad*^−/−^ MEFs in a dose-dependent manner (Fig. 1c and Supplementary information, Figure S1h). More importantly, when the same cytotoxic dose was used, different fractions of the TNFα preparation induced similar degree of apoptosis (Fig. 1d and Supplementary information, Figure S1i). These results demonstrate that the ability of cytotoxic dose TNFα by itself to induce apoptosis is not the result of the sensitization by some unknown pro-apoptotic agent(s) that might be contaminated in the TNFα preparation. In support of this conclusion, TNFα from two other commercial sources (GeneScript and PeproTech) also induced apoptosis in a dose- and BAD-dependent manner, albeit with different potency (Fig. 1e). This was because TNFα from different commercial sources has different biological activities. When similar biological activity was used, i.e., 80 ng/ml of TNFα from R&D, 500 ng/ml of TNFα from GeneScript and 1000 ng/ml of TNFα from PeproTech, TNFα from all three different commercial sources induced comparable BAD-dependent apoptosis (~50–60%) without blocking the IKK signaling pathway (Fig. 1e, Supplementary information, Table S4). Thus, it is the biological activity of TNFα, rather than its dose values, determines whether TNFα by itself is able to induce BAD-dependent apoptosis.

To understand how BAD is involved in cytotoxic dose TNFα-induced apoptosis, we compared the effect of non-cytotoxic and cytotoxic dose TNFα on BAD mitochondrial translocation. Non-cytotoxic dose TNFα did not induce detectable BAD translocation from the cytosol (containing the cytoskeleton) to mitochondria in WT primary hepatocytes, as analyzed by subcellular fractionation followed by immunoblotting (Fig. 1f and Supplementary information, Figure S1j). By contrast, cytotoxic dose TNFα induced a significant amount of BAD to translocate to mitochondria in WT primary hepatocytes (Fig. 1f and Supplementary information, Figure S1j). Similar results were obtained in WT fibroblasts, as analyzed by subcellular fractionation followed by immunoblotting (Supplementary information, Figure S1k) or immunofluorescence (Supplementary information, Figure S1l and S1m). Again, when the same cytotoxic dose was used, different fractions of the TNFα preparation induced similar level BAD mitochondrial translocation (Fig. 1g). This is not the result of defective IKK-mediated phosphorylation of BAD, as cytotoxic dose TNFα-induced IKK-phosphorylated BAD in the cytosol was actually doubled than non-cytotoxic dose TNFα-induced (Fig. 1f, lane 5 compared to lane 3, 18% vs. 8%, 4 h). In support of this notion, in a shorter activation kinetic analysis, cytotoxic and non-cytotoxic dose TNFα induced comparable IKK activation, as evidenced by phosphorylation of IKKβ itself and phosphorylation of its substrates IκBα and BAD (Fig. 1h). Consistently, NF-κB activation was also comparable, as evidenced by similar induction of NF-κB target genes such as cIAP2, IκBα and IL-6 (Fig. 1i). These results further exclude the possibility that some unknown pro-apoptotic agents might be contaminated in the TNFα preparation to sensitize apoptosis by blocking the IKK signaling pathway. Taken together, these results demonstrate that cytotoxic dose TNFα by itself is sufficient to induce apoptosis through promoting BAD mitochondrial translocation despite concurrent activation of IKK and NF-κB.

### IKK is unable to phosphorylate all cytosolic BAD in response to cytotoxic dose TNFα

**Fig. 2:**
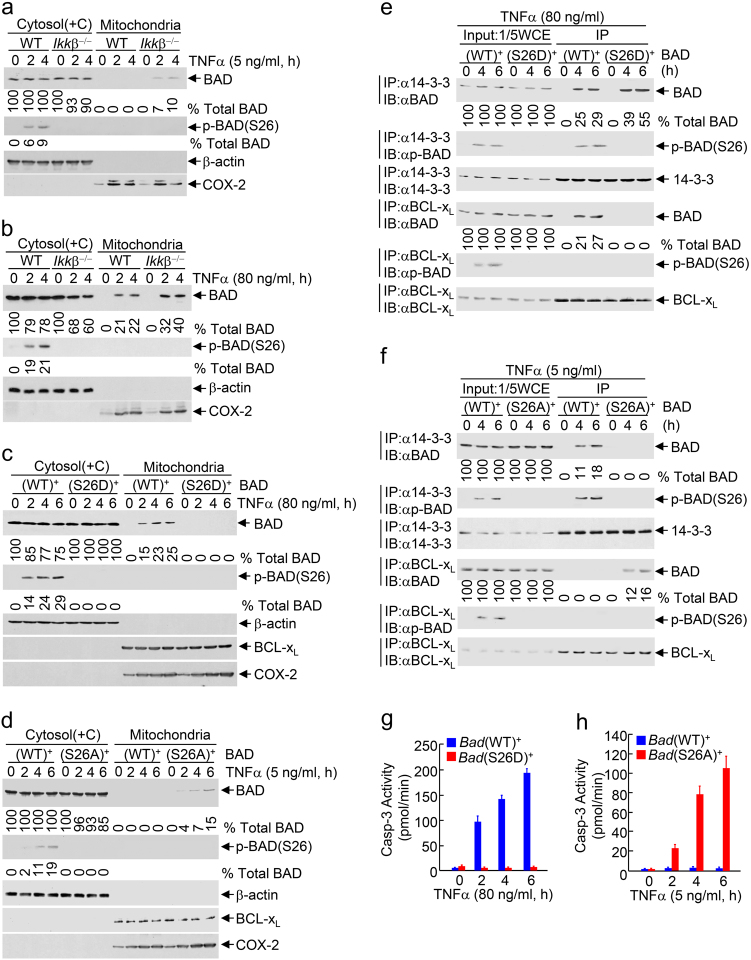
IKK is unable to phosphorylate all cytosolic BAD in cytotoxic dose TNFα-treated MEFs. **a**, **b** WT and *Ikk*β^−/−^ fibroblasts were treated without or with non-cytotoxic dose (**a**) or cytotoxic (**b**) dose TNFα for various durations, as indicated. BAD Ser26-phosphorylation and BAD mitochondrial translocation were determined and quantitated as described in Fig. 1f (Supplementary information, Figure S9c-S9d). **c**, **e**, **g** *Bad*^−/−^ fibroblasts ectopically expressing WT BAD or the BAD(S26D) mutant were treated without or with cytotoxic dose TNFα for various durations, as indicated. BAD phosphorylation and BAD mitochondrial translocation (**c**, Supplementary information, Figure S9e) were determined and quantitated as described in Fig. 1f. The association between BAD WT (or mutant) and 14-3-3 (or BCL-x_L_) were analyzed by immunoprecipitation with anti-14-3-3 or anti-BCL-x_L_ antibody, followed by immunoblotting, respectively. The percentage of immunoprecipitated BAD in total BAD (Input) was quantitated by the ImageJ program (**e**). Apoptotic cells were determined as described in Fig. 1a and represented as means ± s.d. (**g**). **d**, **f**, **h** *Bad*^−/−^ fibroblasts ectopically expressing BAD(WT) or the BAD(S26A) mutant were treated without or with non-cytotoxic dose TNFα for various durations, as indicated. BAD phosphorylation and BAD mitochondrial translocation (**d**, Supplementary information, Figure S9f), the association between BAD WT (or mutant) and 14-3-3 (or BCL-x_L_) (**f**), and apoptotic cells (**h**) were determined as in **c**, **e**, **g**. All data represent two to three individual experiments with similar results

To determine how cytotoxic dose TNFα promotes the pro-apoptotic activity of BAD in the presence of IKK activation, we used WT and *Ikk*β^−/−^ fibroblasts. Upon stimulation by non-cytotoxic dose TNFα, a small amount of non-phosphorylated BAD translocated from the cytosol (containing the cytoskeleton) to mitochondria in *Ikk*β^−/−^ MEFs (Fig. 2a, Upper panel, ~10%, 4 h). Under the same conditions, a similar amount of BAD was phosphorylated by IKK in WT fibroblasts (Fig. 2a, Middle panel, ~9%, 4 h) and there was no detectable BAD mitochondrial translocation (Fig. 2a). This suggests that the cytosolic BAD that translocated to mitochondria in *Ikk*β^−/−^ MEFs was all phosphorylated by IKK in WT fibroblasts. By contrast, in response to cytotoxic dose TNFα, a substantial amount of non-phosphorylated BAD translocated from the cytosol (containing the cytoskeleton) to mitochondria in *Ikk*β^−/−^ MEFs (Fig. 2b, Upper panel, ~40%, 4 h). Under the same conditions, a significant amount of non-phosphorylated BAD also translocated to mitochondria in WT fibroblasts and hepatocytes (Fig. 2b, ~22%, 4 h; Supplementary information, Figure S2a and S2b), even though IKK-phosphorylated BAD was more than doubled than that in non-cytotoxic dose TNFα-treated fibroblasts (Fig. 2b, ~21%, vs. Fig. 2a, ~9%, 4 h; Supplementary information, Figure S2a and S2b; also see Fig. 1f). Note, the sum of phosphorylated and non-phosphorylated BAD in WT fibroblasts was similar to the amount of BAD translocated to mitochondria in *Ikk*β^−/−^ MEFs [Fig. 2b, 43% (22% + 21%) vs. 40%]. Thus, in response to cytotoxic dose TNFα, only about half of cytosolic BAD that translocates to mitochondria in *Ikk*β^−/−^ MEFs is phosphorylated by IKK in WT fibroblasts, resulting in mitochondrial translocation of the remaining non-phosphorylated BAD.

The above observations implicate if all cytosolic BAD were phosphorylated by IKK, cytotoxic dose TNFα should not be able to induce BAD mitochondrial translocation and apoptosis. To test this idea, we used *Bad*^−/−^ MEFs expressing BAD(S26D), in which serine (S) 26 residue was replaced by glutamic acid (D) residue, or BAD(S26A), in which S26 residue was replaced by non-phosphorylatable alanine residue (A). As expected, both BAD(S26D) and BAD(S26A) mutants were no longer phosphorylated by IKK (Fig. 2c, d). Interestingly, BAD(S26D) mutant acted as a phospho-mimetic mutant. Like IKK-phosphorylated WT BAD, BAD(S26D) mutant interacted with 14-3-3 in the cytosol but not BCL-x_L_ on mitochondria in response to cytotoxic dose TNFα (Fig. 2e). Conversely, BAD(S26A) mutant acted as a constitutively active BAD, which did not interact with 14-3-3 in the cytosol but interacted with BCL-x_L_ on mitochondria in response to non-cytotoxic dose TNFα (Fig. 2f). More importantly, cytotoxic dose TNFα was unable to induce mitochondrial translocation of BAD(S26D) mutant (Fig. 2c) and apoptosis (Fig. 2g) in *Bad*(S26D)^+^ MEFs, while even non-cytotoxic dose TNFα was able to induce mitochondrial translocation of BAD(S26A) mutant (Fig. 2d) and apoptosis (Fig. 2h) in *Bad*(S26A)^+^ MEFs. Taken together, although activation of IKK by cytotoxic dose TNFα is even slightly enhanced compared to that by non-cytotoxic dose TNFα, activated IKK was not able to phosphorylate all cytosolic BAD in cytotoxic dose TNFα-treated WT fibroblasts and subsequently non-phosphorylated BAD translocates to mitochondria to trigger apoptosis.

### Cytotoxic dose TNFα induces substantial BAD release from the cytoskeleton to the cytosol by promoting massive depolymerization of actin stress fibers

**Fig. 3:**
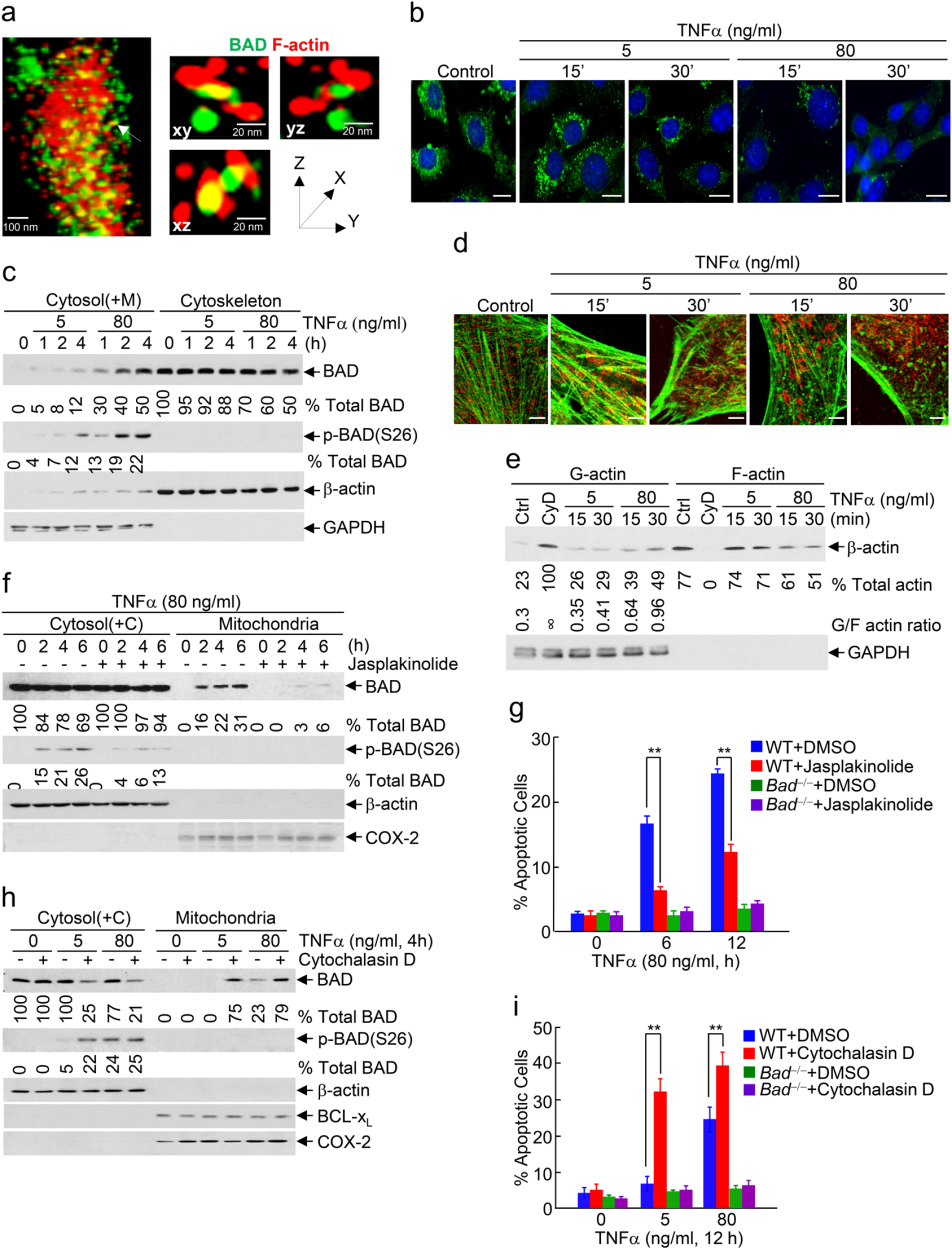
Cytotoxic dose TNFα induces substantial BAD release from the cytoskeleton to the cytosol by promoting massive depolymerization of actin stress fibers. **a** Direct interaction between BAD and F-actin in actin stress fibers at the cytoskeleton was detected by super-resolution microscopy based on Ground State Depletion program. Actin stress fibers were stained with phalloidin**-**Green (Green color). BAD (Red color) was detected by immunofluorescence staining with anti-BAD antibody. Scale bar, 20 nm. **b**, **c** Cytotoxic dose TNFα significantly reduced the interaction of BAD with F-actin, as revealed by proximity ligation assay. Scale bar, 5 μm (**b**), and induced BAD release from the cytoskeleton to the cytosol, as determined by the cytosol [containing mitochondria, Cytosol(+M)] and cytoskeleton fractionation (see “Materials and methods” for details) (Supplementary information, Figure S9g) (**c**). **d** Cytotoxic dose TNFα induced massive depolymerization of actin stress fibers, as detected by double immunofluorescence staining. BAD, Red color; Actin, Green color. Scale bar, 1 μm. **e** WT fibroblasts were stimulated without or with cytotoxic or non-cytotoxic dose TNFα for various durations, as indicated. Cytosol [containing mitochondria, Cytosol(+M)] and cytoskeleton fractions were separated by ultracentrifugation. G-actin and F-actin were detected by immunoblotting with anti-β-actin antibody. Cells treated with Cytochalasin D (CyD; 1 μg/ml; 1 h) were used as positive control. ∞, infinity. GAPDH was used as cytosol marker. The results were quantitated by the ImageJ program. **f**–**i** WT and *Bad*^−/−^ fibroblasts were pre-treated with DMSO or Jasplakinolide (30 nM) (**f**, **g**), Cytochalasin D (1 μg/ml) (**h**, **i**) for 1 h, followed by stimulation without or with cytotoxic or non-cytotoxic dose TNFα for various durations, as indicated. Phosphorylated BAD and BAD mitochondrial translocation were determined and quantitated as described in Fig. 1f (**f**, **h**, Supplementary information, Figure S9h-S9i). Apoptotic cells were determined as described in Fig. 1a. Data are means ± s.d. **P < 0.01, as analyzed by two-tailed unpaired Student’s *t*-test (**g**, **i**). All data represent two to three individual experiments with similar results

We hypothesized that cytotoxic dose TNFα may induce substantial re-distribution of BAD from some unknown subcellular compartment(s) to the cytosol, so that the amount of cytosolic BAD becomes more than IKK can phosphorylate and subsequently non-phosphorylated BAD translocates to mitochondria to trigger apoptosis. To test this scenario, we used super-resolution microscopy based on Ground State Depletion (GSD) program, which is able to detect 3D co-localization between single molecules at a distance ~20 nm.^51, 52^ GSD microscopy revealed that BAD directly interacted with F-actin, the insoluble filament form of actin, in the actin stress fibers at the cytoskeleton in resting WT fibroblasts (Fig. 3a and Supplementary information, Figure S3a), revealing a previously unknown subcellular reservoir for BAD. The actin filament (F-actin) is polymerized from the soluble actin monomer (G-actin) and in turn 10–30 actin filaments (F-actin) bundle along the length and are reversibly cross-linked to form the actin stress fibers. The actin stress fibers undergo polymerization and depolymerization under different condition or stimulation. Since the stress fibers, big actin filaments and the binding proteins can be separated from depolymerized free soluble actin monomer (G-actin), small filaments and the other cytosol proteins by ultracentrifugation (100,000 × *g*), we used sedimentation equilibrium, an analytic method that can detect protein complex by ultracentrifugation, to further analyze the interaction between BAD and the actin stress fibers at the cytoskeleton. Consistently, BAD interacted with the insoluble filament F-actin in the actin stress fibers but not the free soluble G-actin monomer in the solution (Supplementary information, Figure S3b and S3c), suggesting that the interaction of BAD with F-actin is facilitated by polymer actin stress fibers. Furthermore, proximity ligation assay, which has a resolution for protein-protein interaction at <40 nm,^53, 54^ demonstrated that BAD selectively interacted with F-actin in actin stress fibers at the cytoskeleton (Fig. 3b and Supplementary information, Figure S3d), but not Tubulin or Vimentin, which are markers of microtubules and intermediate filament at the cytoskeleton, respectively (Supplementary information, Figure S3e and S3f). More importantly, the interaction of BAD with F-actin was rapidly and significantly reduced by cytotoxic dose TNFα (Fig. 3b and Supplementary information, Figure S3g). Cell fractionation assay also showed that non-cytotoxic dose TNFα only induced modest BAD release from the cytoskeleton to the cytosol (Fig. 3c, Upper panel, ~12% at 4 h), in which all BAD was phosphorylated by IKK (Fig. 3c, Middle panel). By contrast, cytotoxic dose TNFα induced rapid and substantial BAD release from the cytoskeleton to the cytosol (Supplementary information, Figure S3h; Fig. 3c, Upper panel, ~50% at 4 h), in which less than half of BAD was phosphorylated by IKK (Fig. 3c, Middle panel, ~22% at 4 h) and the rest of BAD was not phosphorylated (Fig. 3c). Interestingly, cytotoxic dose TNFα induced similar levels of WT BAD, BAD(S26D) and BAD(S26A) release from the cytoskeleton to the cytosol (Supplementary information, Figure S3i), suggesting that BAD release from the cytoskeleton is independent on its phosphorylation state. Thus, in resting cells BAD is sequestered by its interaction with F-actin in actin stress fibers at the cytoskeleton, the previous unknown reservoir for BAD, and cytotoxic dose TNFα induces substantial BAD release from the cytoskeleton, resulting in significantly increased amount of BAD in the cytosol.

To determine the mechanism by which cytotoxic dose TNFα induces substantial BAD release from the cytoskeleton to the cytosol, we analyzed the effect of TNFα on the actin stress fiber stability. Confocal fluorescence microscopy revealed that cytotoxic dose TNFα induced significant depolymerization of actin stress fibers (Fig. 3d; Supplementary information, Movie S1 and S2). Consistently, the ratio of G-actin to F-actin, which is an index of actin stress fiber depolymerization, was significantly increased by cytotoxic dose TNFα (Fig. 3e). Cytotoxic dose TNFα-induced BAD mitochondrial translocation and apoptosis were significantly reduced by actin stress fiber polymerization stabilizers such as jasplakinolide or sphingosine-1-phosphate (Fig. 3f, g, Supplementary information, Figure S4a and S4b). Conversely, non-cytotoxic dose TNFα was able to induce BAD mitochondrial translocation and apoptosis in the presence of actin stress fiber polymerization inhibitors like cytochalasin D or latrunculin B (Fig. 3h, i, Supplementary information, Figure S4c and S4d). Taken together, these results demonstrate that cytotoxic dose TNFα induces massive depolymerization of actin stress fibers, thereby promoting substantial BAD release from the cytoskeleton to the cytosol to overcome IKK-mediated inhibition.

### Cytotoxic dose TNFα induces significant early-phase inactivation of RhoA through preferential activation of the Src-p190GAP pathway

**Fig. 4:**
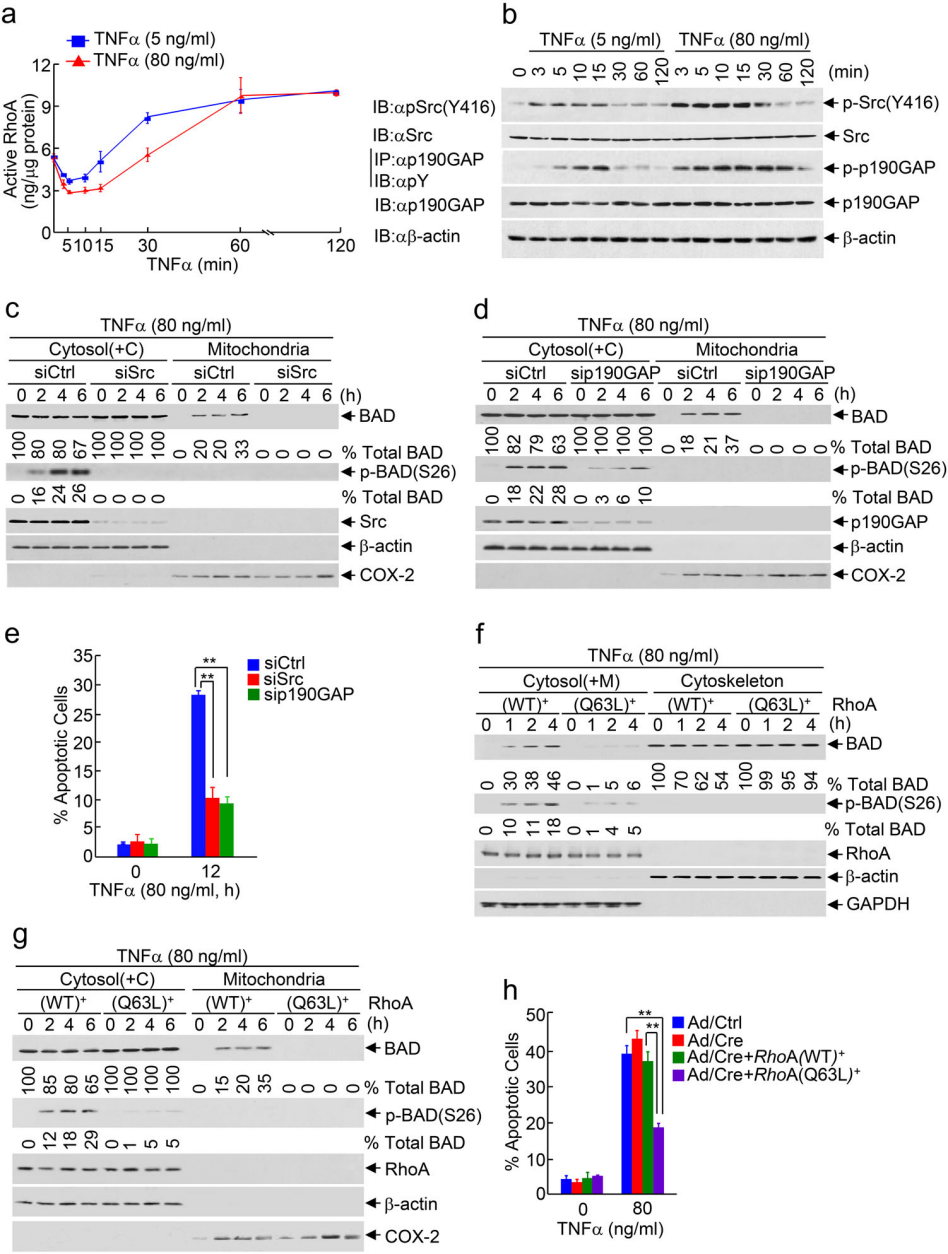
Cytotoxic dose TNFα induces massive depolymerization of actin stress fibers through Src-p190GAP-mediated inactivation of RhoA. **a** Cytotoxic dose TNFα induced stronger and sustained early-phase inactivation and delayed activation of RhoA than non-cytotoxic dose TNFα, as measured by the G-LISA RhoA activation assay. Data are means ± s.d. **b** Cytotoxic dose TNFα induced stronger and sustained activation of Src and p190GAP than non-cytotoxic dose TNFα. Phosphorylated p190GAP was detected by immunoprecipitation with anti-p190GAP antibody and immunoblotting with anti-phosphor-Tyrosine (αpY) antibody. **c**–**e** WT fibroblasts were transfected with scramble siRNA (siCtrl) (**c**–**e**), siSrc (**c**, **e**), or sip190GAP (**d**, **e**), followed by treatment without or with cytotoxic dose TNFα for various durations, as indicated. BAD mitochondrial translocation and IKK-phosphorylated BAD were determined and quantitated as described in Fig. 1f (**c**, **d**, Supplementary information, Figure S9j-S9k). **e** Apoptotic cells were determined as described in Fig. 1a. **f**–**h** Primary *Rho*A^*f/f*^ fibroblasts isolated from *Rho*A^*f/f*^ mice were infected with Ad/Ctrl or Ad/Cre (10 multiplicity of infection, moi) and transfected with or without expressing vector pcDNA3 encoding WT or the Q63L mutant RhoA, followed by treatment without or with cytotoxic dose TNFα for various durations, as indicated. Ectopically expressed RhoA(Q63L) mutant but not WT RhoA inhibited cytotoxic dose TNFα-induced BAD release from the cytoskeleton (**f**, Supplementary information, Figure S9l), mitochondrial translocation (**g**, Supplementary information, Figure S9m), and apoptosis (**h**). Data in **e**, **h** are means ± s.d. **P < 0.01, as analyzed by two-tailed unpaired Student’s *t*-test. All data represent two to three individual experiments with similar results

We reasoned that cytotoxic dose TNFα may induce depolymerization of actin stress fibers through inactivation of RhoA, which is a member of Rho subfamily GTPase that plays a crucial role in formation of actin stress fibers at the cytoskeleton.^55^ Although RhoA was reported to be activated by TNFα through transactivation of EGFR signaling,^56^ a detailed kinetic analysis of RhoA activation revealed that TNFα regulated RhoA in a biphasic manner with an early-inactivation phase (5–30 min post stimulation) and a late-activation phase (30 min post stimulation) (Fig. 4a). In comparison to non-cytotoxic dose TNFα, cytotoxic dose TNFα-induced early-inactivation of RhoA was stronger and sustained, accompanying with a much slower late-phase activation of RhoA (Fig. 4a), correlating to BAD release from the cytoskeleton to the cytosol, which occurred as early as 15 min post stimulation (Supplementary information, Figure S3h). The differential regulation of RhoA by non-cytotoxic and cytotoxic dose TNFα was specific, since there was no detectable difference between non-cytotoxic and cytotoxic dose TNFα in activation of another Rho subfamily GTPase member Cdc42 (Supplementary information, Figure S5a), which regulates actin polymerization at the plasma membrane.^57^

RhoA activity is inactivated by a specific Rho GTPase activating protein (p190GAP),^58^ whose activity is stimulated by Src-mediated tyrosine phosphorylation,^59^ but is activated by GTPase exchange factor H1 (GEF-H1), whose activity is stimulated by ERK.^56^ TNFα is known to induce Src(Y416) autophosphorylation and activation.^60^ Consistently, cytotoxic dose TNFα induced much more profound phosphorylation of Src at tyrosine416 than non-cytotoxic dose TNFα (Fig. 4b and Supplementary information, S5b), resulting in stronger and prolonged Src-mediated tyrosine phosphorylation of p190GAP (Fig. 4b and Supplementary information, Figure S5c). By contrast, cytotoxic and non-cytotoxic dose TNFα induced comparable ERK activation (Supplementary information, Figure S5d and S5e) and GEF-H1 activation (Supplementary information, Figure S5f). These results suggest that preferential activation of the Src-p190GAP pathway by cytotoxic dose TNFα leads to stronger and sustained early-phase inactivation of RhoA. In support of this notion, knockdown of Src or p190GAP reduced cytotoxic dose TNFα-induced depolymerization of actin stress fibers, as measured by G/F actin ratio (Supplementary information, Figure S5g and S5h). Taken together, cytotoxic dose TNFα significantly inactivates RhoA through preferential activation of the Src-p190GAP pathway, resulting in massive depolymerization of actin stress fibers to promote substantial BAD release from the cytoskeleton to the cytosol.

### Inactivation of RhoA by the Src-p190GAP pathway is responsible for cytotoxic dose TNFα-induced apoptosis

To determine whether inactivation of RhoA by the Src-p190GAP pathway, which leads to massive depolymerization of actin stress fibers at the cytoskeleton, is responsible for cytotoxic dose TNFα-induced BAD mitochondrial translocation and apoptosis, we used WT fibroblasts expressing siRNA against Src or p190GAP. Immunoblotting analysis revealed that cytotoxic dose TNFα-induced BAD mitochondrial translocation was completely blocked by silence of Src (Fig. 4c) or p190GAP (Fig. 4d), so was cytotoxic dose TNFα-induced apoptotic cell death (Fig. 4e).

To definitively demonstrate that cytotoxic dose TNFα-induced apoptosis is mediated by inactivation of RhoA, but not other cellular activities, we used the constitutively active RhoA(Q63L) mutant, in which the replacement of glutamine (Q) 63 residue by leucine (L) residue abolishes RhoA GTPase activity and thereby renders it resistant to inhibition by GTPase GAP such as p190GAP.^61^ Indeed, ectopic expression of RhoA(Q63L) mutant but not WT RhoA in *Rho*A^−/−^ MEFs significantly inhibited cytotoxic TNFα-induced substantial BAD release from the cytoskeleton (Fig. 4f), BAD mitochondrial translocation (Fig. 4g) and apoptotic cell death (Fig. 4h). Taken together, these results demonstrate that cytotoxic dose TNFα induced BAD mitochondrial translocation and apoptosis depends on Src-p190GAP-mediated early-phase inactivation of RhoA.

### Loss of BAD protects mice from septic shock-induced mortality and preserves the organ integrity in septic shock

**Fig. 5:**
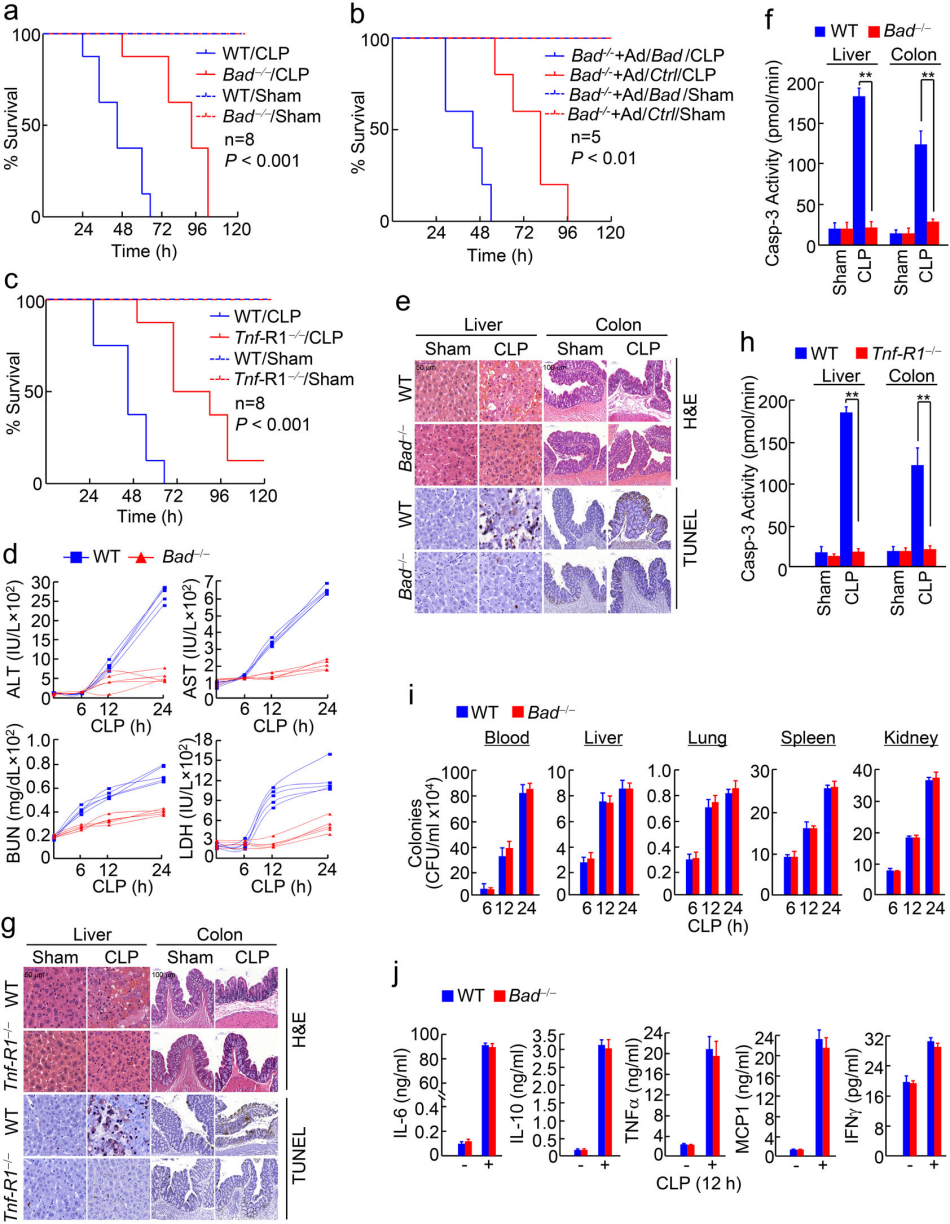
Loss of Bad protects mice from septic shock-induced tissue damage of multiple organs and mortality. **a** WT littermates and *Bad*^−/−^ mice were subjected to high-grade cecal ligation and puncture (CLP) surgery or Sham procedure (see “Materials and methods” for details) and the mortality rate was determined. *P* < 0.001; *n* = 8, as analyzed by log-rank (Mantel–Cox) test. **b** *Bad*^−/−^ mice were injected intravenously Ad/*Bad* and Ad/*Ctrl* (LacZ) (see “Materials and methods” for details) and then subjected to high-grade CLP surgery or Sham procedure, as described in **a**. The mortality rate was determined. *P* < 0.01; *n* = 5, as analyzed by log rank (Mantel–Cox) test. **c**–**j** *Bad*^−/−^ mice and their WT littermates (**d**–**f**, **i**, **j**) or *Tnf*-R1^−/−^ mice and their WT littermates (**c**, **g**, **h**) were subjected to high-grade CLP surgery or Sham procedure, as described in **a**. **c** The mortality rate was determined. *P* < 0.001; *n* = 8, as analyzed by log-rank (Mantel–Cox) test. **d** Blood samples at different time points as indicated were collected through tail vein. Serum ALT, AST, BUN and LDH were determined. **e**, **g** Mice underwent high-grade CLP or Sham surgery for 24 h were pre-removed to collect the liver and colon tissues for hematoxylin and eosin (H&E) staining respectively. **f**, **h** Caspase 3 activity in liver and colon tissue samples were analyzed. **P < 0.01, as determined by two-tailed unpaired Student’s *t*-test. **i** Bacteria load in the circulation or certain tissues was analyzed (see “Materials and methods” for details). **j** Concentrations of IL-6, IL-10, TNFα, MCP1, and IFNγ in the circulation were detected by Cytometric Bead Array (see “Materials and methods” for details). Data in **f**, **h**–**j** are presented as means ± s.d. and represent three individual experiments

To determine whether BAD mediates TNFα cytotoxicity in disease, we used the cecal ligation and puncture (CLP) murine model of septic shock,^62^ which replicates the septic shock in human patients better than other murine models.^63^ In this model, polymicrobial infection of abdomen causes bacteremia and overly systemic inflammatory response, the latter of which is responsible for tissue damage of multiple organs and mortality,^2–4^ in which TNFα cytotoxicity is known to have a crucial role.^25–28^ Under the conditions of the high-grade sepsis induced by CLP, WT littermates started to die around 24 h (Fig. 5a), as reported previously.^62^ By contrast, *Bad*^−/−^ mice were significantly protected and only started to die around 48 h (Fig. 5a). Reconstitution of *Bad*^−/−^ mice with WT Bad via adenovirus infection restored the sensitivity of *Bad*^−/−^ mice to polymicrobial infection (Fig. 5b), indicating that loss of Bad, but not other potential genetic alterations in *Bad*^−/−^ mice, is responsible for the protection against polymicrobial infection. Importantly, *Tnf-*R1^−/−^ mice had significantly reduced mortality than their WT littermates (Fig. 5c), demonstrating that polymicrobial infection induces the mortality in a TNFα signaling-dependent manner, consistent with the previous report.^28^ Similar results were obtained with lethal dose LPS, a structural component of the outer membranes of Gram-negative bacteria that is able to induce septic shock by triggering overly production of TNFα and other pro-inflammatory cytokines in the absence of bacteria (Supplementary information, Figure S6a).^29^

Next, we determined the role of Bad in tissue damage of multiple organs in septic shock. Under conditions of CLP-induced high-grade sepsis, WT littermates but not *Bad*^−/−^ mice had significantly increased serum concentrations of several tissue damage markers including ALT (liver), AST (liver, heart and muscle), BUN (kidney), and LDH (lung and general cellular damage) (Fig. 5d), suggesting that loss of Bad protects organs from tissue damage in septic shock. This notion was further supported by the observation that while WT littermates had profound tissue damage of liver, as evidenced by loss of hepatocytes, hemorrhaging and neutrophil infiltration, and colon, as evidenced by epithelium erosion, loss of crypt and goblet cells and neutrophil infiltration 12 h post-CLP, *Bad*^−/−^ mice were resistant to polymicrobial infection-induced tissue damage of liver and colon (Fig. 5e). The tissue damage was accompanied with massive apoptosis, as analyzed by TUNEL assay (Fig. 5e) and Casp-3 activity assay (Fig. 5f). *Tnf-*R1^−/−^ mice were also significantly protected from polymicrobial infection-induced tissue damage of liver and colon (Fig. 5g, h), suggesting that polymicrobial infection induces tissue damage of liver and colon in a TNFα signaling-dependent manner, consistent with previous reports.^28^ Similar results were obtained in LPS-treated *Bad*^−/−^ mouse liver and colon (Supplementary information, Figure S6b-S6d). Taken together, BAD is responsible for the loss of tissue integrity of liver and colon, and likely other target organs as well, thereby contributing to the mortality in septic shock.

### Loss of Bad confers disease tolerance to septic shock

The above observations promoted us to determine whether loss of Bad confers disease tolerance^64–66^ to septic shock. Under conditions of CLP-induced high-grade sepsis, *Bad*^−/−^ mice and their WT littermates had similar bacterial load in blood and target organs of septic shock, such as liver, spleen, kidney, and lung (Fig. 5i). Under the same conditions, *Bad*^−/−^ mice and their WT littermates had dramatically increased but comparable cytokine production, as evidenced by protein levels of pro- and anti-inflammatory cytokines including TNFα, IL-6, MCP-1, IL-10, and IFNγ in blood (Fig. 5j), as well as the transcriptional levels of TNFα, IL-1β, IL-6, IL-10, and IFNγ in liver and colon (Supplementary information, Figure S6e). The profiles of cytokine expression and production suggest that neither pro- nor anti-inflammatory cytokines was affected by loss of BAD. Similar results were obtained with lethal dose LPS-treated *Bad*^−/−^ mice and their WT littermates (Supplementary information, Figure S6f). These results demonstrate that loss of BAD confers disease tolerance to septic shock without directly affecting bacterial burden or inflammatory response, consistent with previous reports that TNFα impairs disease tolerance in several infectious diseases.^67–69^

### Polymicrobial infection stimulates BAD pro-apoptotic activity in septic shock

**Fig. 6:**
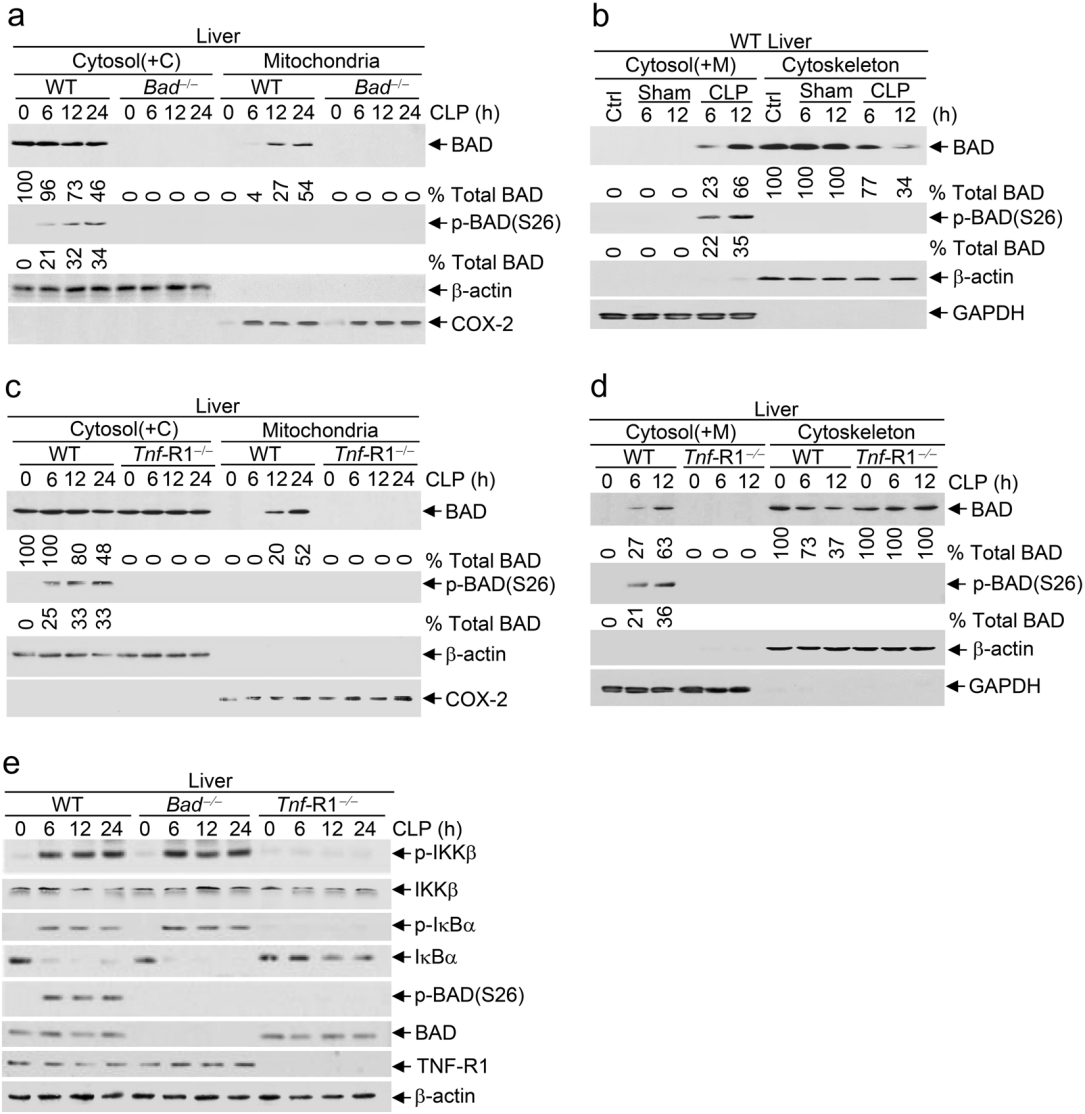
Polymicrobial infection activates BAD pro-apoptotic activity in septic shock. *Bad*^−/−^ mice and their WT littermates (**a**, **b**, **e**), or *Tnf*-R1^−/−^ mice and their WT littermates (**c**–**e**) were subjected to high-grade CLP surgery or Sham procedure, as described in Fig. 5a. **a**–**d** Total liver tissue extracts were fractionated to determine BAD translocation from the cytosol [(containing cytoskeleton, Cytosol(+C)] to mitochondria (see “Materials and methods” for details) (**a**, **c** and Supplementary information, Figure S9n, S9p), or BAD release from the cytoskeleton to the cytosol [containing mitochondria; Cytosol(+M)] (see “Materials and methods” for details) (**b**, **d** and Supplementary information, Figure S9o, S9q). The levels of BAD and the percentage of IKK-phosphorylated BAD in total BAD were determined and quantitated, as described in Fig. 1f (See “Materials and methods” for details). **e** Phosphorylation of IKKβ, IκBα, phospho-BAD(S26), as well as expression levels of IKKβ, IκBα, BAD, TNF-R1, and β-actin in liver were analyzed by immunoblotting with corresponding antibodies. All the results represent three individual experiments with similar results

We wondered whether polymicrobial infection stimulates BAD pro-apoptotic activity. Under conditions of CLP-induced high-grade sepsis, a significant amount of non-phosphorylated BAD translocated to mitochondria in the liver of WT littermate (Fig. 6a, ~27%, 12 h), even though a similar amount of BAD was simultaneously phosphorylated by IKK at Ser26 (Fig. 6a, 32%, 12 h). Under the same conditions, a substantial amount of BAD was released from the cytoskeleton to the cytosol (Fig. 6b, Upper panel, ~66%; 12 h), only about half of which was phosphorylated by IKK (Fig. 6b, Middle panel, ~35%, 12 h). These results demonstrate that like cytotoxic dose TNFα, polymicrobial infection induces much more BAD release from the cytoskeleton to the cytosol than IKK can phosphorylate, so that the remaining non-phosphorylated BAD translocates to mitochondria to induce apoptosis. This notion is further supported by the observation that polymicrobial infection-induced BAD release from the cytoskeleton and mitochondrial translocation were completely blocked in the liver of *Tnf-*R1^−/−^ mice (Fig. 6c, d), indicating that polymicrobial infection stimulates BAD pro-apoptotic activity in a TNFα-dependent manner. Like cytotoxic dose TNFα, polymicrobial infection induced comparable IKK activation in the livers of WT littermate and *Bad*^−/−^ mice, but not in the livers of *Tnf*-R1^−/−^ mice as expected (Fig. 6e), further suggesting that stimulation of the BAD pro-apoptotic activity by polymicrobial infection is not the result of defective IKK activation. Similar results were obtained with lethal dose LPS (Supplementary information, Figure S7a-S7c). Taken together, these results demonstrate that polymicrobial infection utilizes the same mechanism as cytotoxic dose TNFα to stimulate BAD pro-apoptotic activity in septic shock.

### Elimination of BAD pro-apoptotic activity reduces mortality and suppresses tissue damage of liver and colon in septic shock

**Fig. 7:**
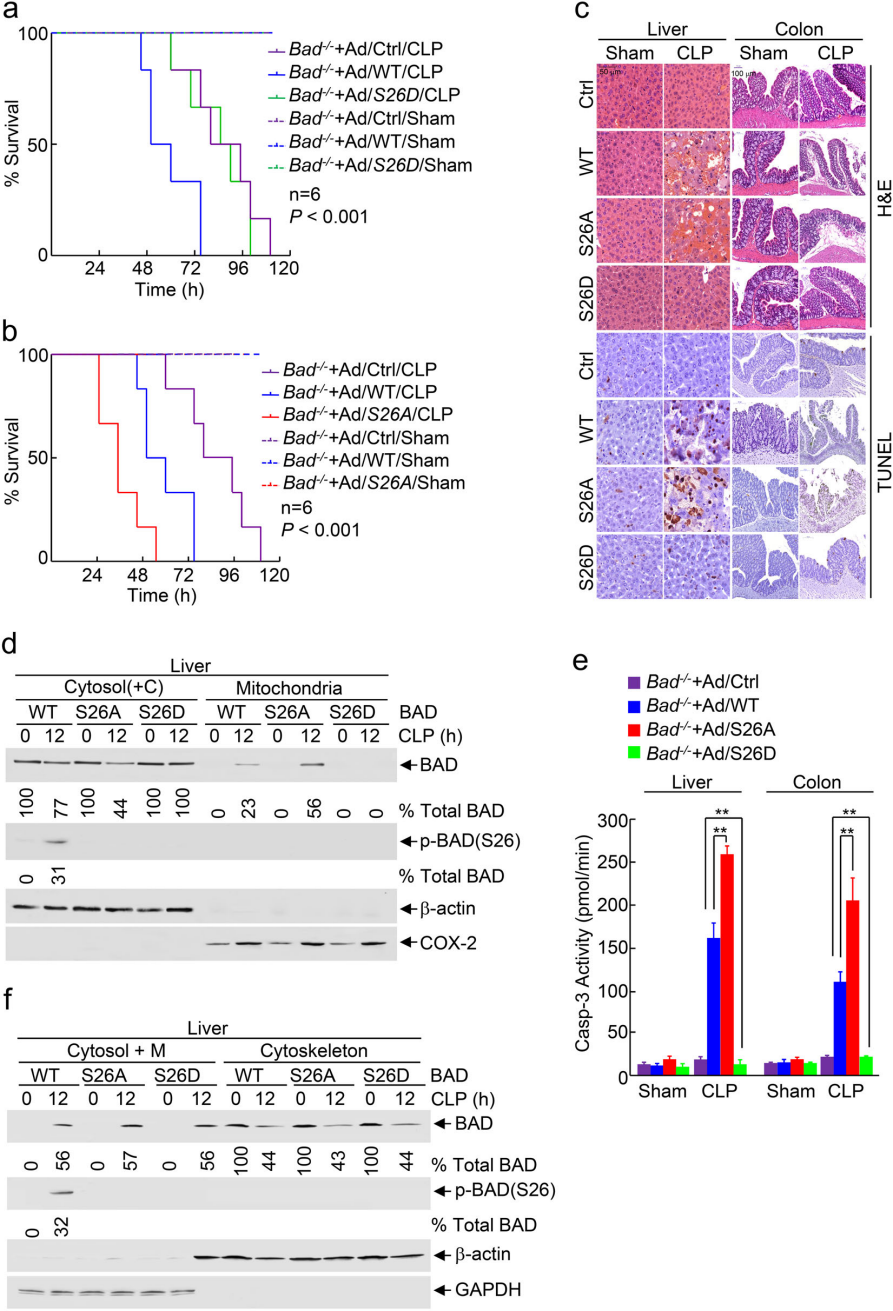
Elimination of BAD pro-apoptotic activity reduces mortality rate and suppresses tissue damage of liver and colon in septic shock. *Bad*^−/−^ mice were intravenously injected with Ad/WT, Ad/S26A, Ad/S26D BAD, or the control Ad/Ctrl (LacZ), respectively, for 48 h and then subjected to high-grade cecal ligation and puncture (CLP) surgery or Sham procedure. **a**, **b** The mortality rate was determined. *P* < 0.001; *n* = 6, as analyzed by log-rank (Mantel–Cox) test. **c** Liver and colon tissues from pre-removed mice 24 h post-CLP were analyzed by H&E staining and TUNEL staining. **d**, **f** Total liver tissue extracts were fractionated to determine BAD translocation from the cytosol [contain the cytoskeleton, Cytosol(+C)] to mitochondria (**d**, Supplementary information, Figure S9r), or BAD release from the cytoskeleton [contain mitochondria, cytosol(+C)] to the cytosol (**f**, Supplementary information, Figure S9s) at 0 and 12 h post-CLP. The levels of BAD and the percentage of IKK-phosphorylated BAD in total BAD were determined and quantitated, as described in Fig. 1f (See “Materials and methods” for details). The results represent three individual experiments with similar results. **e** Caspase 3 activity in liver and colon tissues isolated from Sham or 24 h post-CLP mice was analyzed, **P < 0.01, as analyzed by two-tailed unpaired Student’s *t*-test

**Fig. 8:**
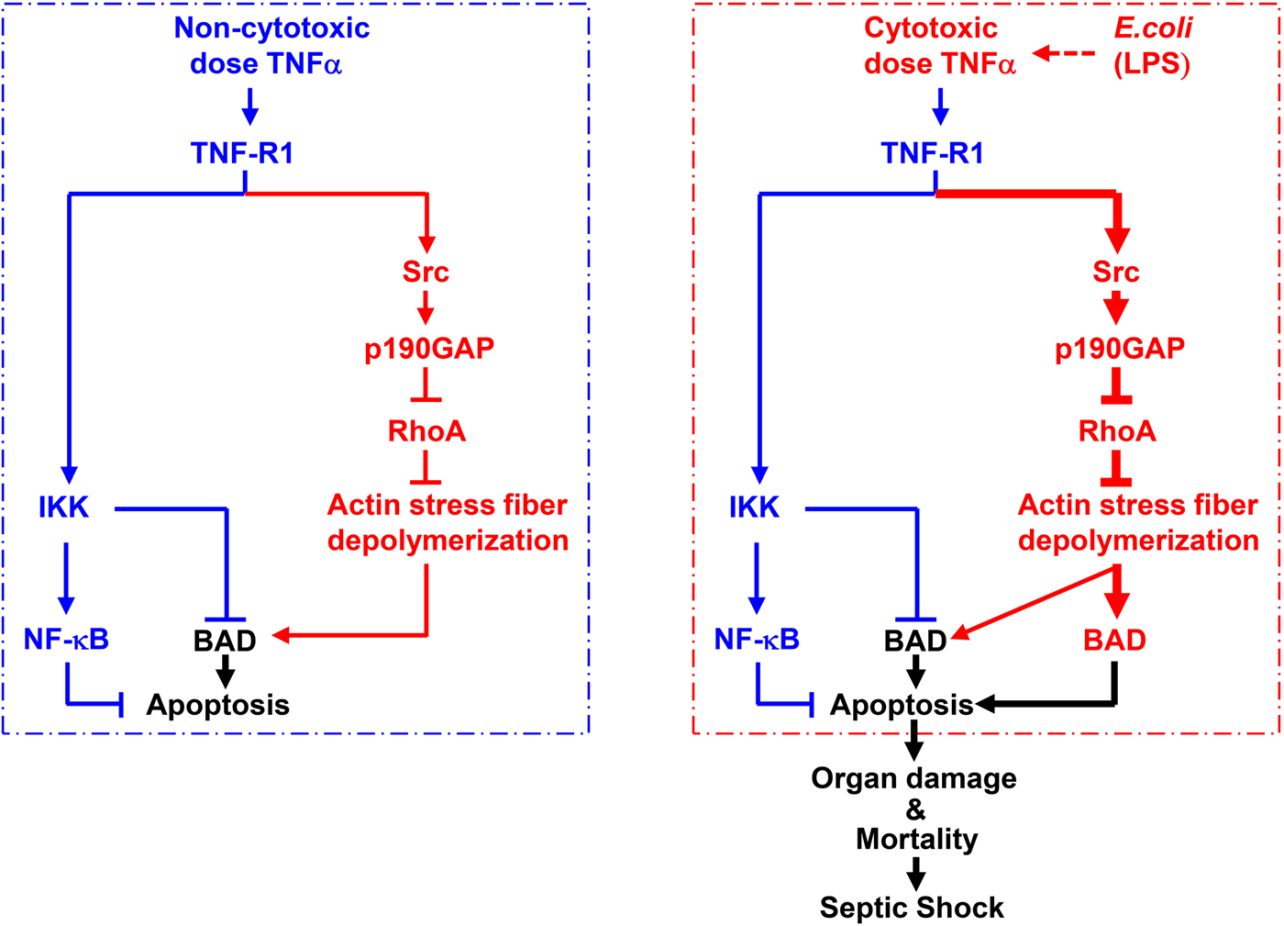
A schematic presentation of the mechanism underlying TNFα cytotoxicity in vivo. Left panel, non-cytotoxic dose TNFα-induced apoptosis is typically suppressed by IKK-mediated activation of NF-κB and inactivation of BAD.^14^ Under this condition, non-cytotoxic dose TNFα does induce limited BAD released from the cytoskeleton to the cytosol via transient and modest activation of the Src-p190GAP-RhoA pathway and subsequently all cytosolic BAD is phosphorylated and inactivated by IKK, thereby there is no apoptosis. Right panel, although cytotoxic dose TNFα induces slightly higher level activation of IKK, which is still capable of activating NF-κB and inactivating BAD to inhibit apoptosis, it induces massive BAD release from the cytoskeleton to the cytosol through prolonged and strong activation of the Src-p190GAP-RhoA pathway. Under this condition, the amount of BAD in the cytosol becomes much more than IKK can phosphorylate. Consequently, non-phosphorylated BAD translocates to mitochondria to induce apoptosis despite concurrent activation of the IKK-NF-κB pathway. Polymicrobial infection and LPS utilize the same mechanism as cytotoxic dose TNFα to induce septic shock. The red arrows were thicker in Right panel than the ones in Left panel, indicating that cytotoxic dose TNFα induces prolonged and strong activation of the Src-p190GAP-RhoA pathway than non-cytotoxic dose TNFα. See the text for additional details

To determine that BAD pro-apoptotic activity is involved in polymicrobial infection-induced cell death, tissue damage of multiple organs and mortality in septic shock, we used *Bad*^−/−^ mice that have been reconstituted through adenoviral vector encoding WT Bad [*Bad*(WT)^+^], the phospho-mimetic Bad(S26D) mutant [Bad(S26D)^+^] (Fig. 2c), or the non-phosphorylatable Bad(S26A) mutant [*Bad*(S26A)^+^] (Fig. 2d). Under conditions of CLP-induced high-grade sepsis, *Bad*(WT)^+^ mice regained the sensitivity to polymicrobial infection-induced mortality, but *Bad*(S26D)^+^ mice remained as insensitive as *Bad*^−/−^ mice (Fig. 7a). By contrast, *Bad*(S26A)^+^ mice were even more sensitive than *Bad*(WT)^+^ mice (Fig. 7b). Consistently, *Bad*(S26D)^+^ mice were resistant while *Bad*(S26A)^+^ mice were more sensitive to polymicrobial infection-induced liver and colon damage that was accompanied with massive apoptosis (Fig. 7c), suggesting that elimination of BAD pro-apoptotic activity protects the mice from polymicrobial infection-induced mortality and liver and colon damage. In support of this notion, BAD mitochondrial translocation and Casp-3 activation in the liver were completely blocked in *Bad*(S26D)^+^ mice but augmented in *Bad*(S26A)^+^ mice (Fig. 7d, e). Note, there was no difference in BAD release from the cytoskeleton to the cytosol between *Bad*(S26D)^+^ and *Bad*(S26A)^+^ mice (Fig. 7f), consistent with the in vitro finding that cytotoxic dose TNFα-induced BAD release from the cytoskeleton to the cytosol is independent of its phosphorylation state (Supplementary information, Figure S3i). The difference between BAD(S26D) and BAD(S26A) mutant in terms of protection of the mice from polymicrobial infection was not the result of variations in Bad construct expressions, as the protein levels of WT and mutant BAD were similar to each other in liver or colon (Supplementary information, Figure S8). Taken together, these results demonstrate that cytotoxic dose TNFα-induced pro-apoptotic activity of BAD has a crucial role in mortality and tissue damage of multiple organs in septic shock and suggest that blocking BAD-mediated TNFα cytotoxicity may have the potential in preserving the organ integrity and reduction of mortality rate in septic shock (Fig. 8).

## Discussion

It has long been recognized that TNFα typically does not induce apoptosis unless IKK-mediated activation of NF-κB and inactivation of BAD are impaired in various cultured cells and genetic animal models.^14, 70^ Paradoxically, there are little known genetic defects in or pharmaceutic inhibitors of the IKK signaling pathway in many inflammatory and infectious diseases, where TNFα cytotoxicity is known to contribute to tissue damage of multiple organs and even mortality. The underlying mechanism is not known. In this report, we demonstrate that BAD mediates TNFα cytotoxicity despite concurrent activation of the IKK signaling pathway in vitro in high-dose TNFα-treated cultured mammalian cells and in vivo in septic shock (Fig. 8), thereby providing a long-sought mechanism for TNFα cytotoxicity in disease.

Cytotoxic dose TNFα is sufficient to induce apoptosis in a variety of mammalian cells despite concurrent activation of the IKK signaling pathway. Whether TNFα by itself is sufficient to induce apoptosis is highly controversy. Previously, it has been shown that TNFα by itself induced cell death in NIH3T3 fibroblasts in a TNF-R1-dependent and dose-dependent manner with no known mechanism.^71^ However, this phenomenon was not observed in some other studies.^72–74^ Our results show that at high doses, TNFα induced apoptosis in a variety of mammalian cells without blocking the IKK signaling pathway (Fig. 1a, b). An obvious concern is whether the TNFα preparation used in our study was inadvertently contaminated by some unknown pro-apoptotic agents that could sensitize the apoptotic activity of TNFα. This is not the case. First, the MS/MS mass spectrometry analysis revealed that the TNFα preparation did not have any detectable chemical modifications or contain any other detectable proteins or chemicals (Supplementary information, Table S1 and S2). Second, different chromatograph fractions of the TNFα preparation were able to induce apoptosis in a dose- and BAD-dependent manner (Fig. 1c, Supplementary information, Figure S1g-S1i). More importantly, when the same cytotoxic dose was used, different fractions of the TNFα preparation with the same amount of stabilizing agent BSA (Supplementary information, Table S3) induced similar degree of apoptosis (Fig. 1d and Supplementary information, Figure S1i). These results definitely exclude the possibility of any contamination, because any unknown contaminant cannot co-elute with TNFα in different chromatographic fractions. A possible explanation for the apparent discrepancy is that the ability of TNFα to induce apoptosis is dependent on its biological activity, not simply based on its dose (Fig. 1e; Supplementary information, Table S4). TNFα with low biological activity was unable to induce apoptosis at the same or even higher dose as TNFα with high biological activity (Fig. 1e). Another possible explanation is that the ability of TNFα to induce apoptosis is affected by NF-κB activity in the target cells. Our results show that cytotoxic dose TNFα induces apoptosis through activation of BAD-mediated mitochondrial death pathway, in which the executioner apoptotic caspases are the known targets of NF-κB-induced inhibitors of apoptosis (IAPs).^75^ Indeed, when pre-treated with non-cytotoxic dose TNFα, which induces NF-κB activation without apoptosis, the cells were protected from cytotoxic dose TNFα-induced apoptosis.^75^ Cells with constitutive activation of NF-κB, such as many tumor cells and some cultured cells, will be resistant to cytotoxic dose TNFα-induced apoptosis. Future studies are needed to test this hypothesis. Thus, the ability of TNFα to induce apoptosis is determined, at least, by its intrinsic biological activity and the cellular context of the target cells.

The cytoskeleton is a previous-unknown BAD reservoir and has a key role in regulation of BAD pro-apoptotic activity. Previously, it has been reported that upon stimulation by non-cytotoxic dose TNFα, BAD is phosphorylated by IKK, which primes BAD to be further phosphorylated by other protein kinases and subsequently sequestered by 14-3-3 in the cytosol, thereby preventing BAD mitochondrial translocation and apoptosis.^14^ Paradoxically, majority of the cytoplasmic BAD is not phosphorylated by IKK or other protein kinases, but it does not translocate to mitochondria to induce apoptosis either.^14^ Our results demonstrate that in resting cells BAD was sequestered by the cytoskeleton by selectively interacting with F-actin of actin stress fibers and was released to the cytosol in response to TNFα (Fig. 3). Non-cytotoxic dose TNFα induced limited depolymerization of actin stress fibers, resulting in modest BAD release from the cytoskeleton to the cytosol, where all BAD was phosphorylated by IKK, further sequestered by 14-3-3 in the cytosol and thereby unable to translocate to mitochondria (Figs. 2 and 3). This explains how non-cytotoxic dose TNFα does not typically induce apoptosis unless activation of the IKK signaling pathway is impaired.^14^ By contrast, cytotoxic dose TNFα induced massive depolymerization of actin stress fibers, leading to substantial BAD release from the cytoskeleton to the cytosol, in which only a fraction of overly increased BAD was phosphorylated by IKK and the remaining non-phosphorylated BAD translocated to mitochondria to induce apoptosis (Figs. 2 and 3). This explains how cytotoxic dose TNFα is able to induce apoptosis despite concurrent activation of the IKK signaling pathway. It is most likely that the failure of IKK to phosphorylate all cytosolic BAD in WT fibroblasts in response to cytotoxic dose TNFα is because the amount of cytosolic BAD is much more than IKK can phosphorylate (Figs. 2 and 3). However, we cannot formally exclude the possibility that in response to cytotoxic dose TNF some unknown temporal-spatial factors might affect the accessibility of BAD proteins to IKK. Future studies are needed to explore this scenario and to determine whether other death stimuli also induce apoptosis by promoting substantial BAD release from the cytoskeleton to the cytosol and subsequent BAD mitochondrial translocation.

Inactivation of RhoA by the Src-p190GAP pathway is essential for cytotoxic dose TNFα-induced apoptosis. The stability of actin stress fibers is determined by the balance between polymerization and depolymerization. Previously, it has been reported that RhoA has a crucial role in promotion of actin stress fiber polymerization,^55^ and the activity of RhoA is inactivated by Src-mediated activation of p190GAP but is activated by ERK-mediated activation of GEF-H1.^56, 58, 59^ Our results demonstrate that TNFα regulated RhoA in a biphasic manner, i.e., an early-inactivation phase followed by a late-activation phase (Fig. 4a). While both non-cytotoxic and cytotoxic dose TNFα induced comparable activation of the ERK-GEF-H1 pathway, cytotoxic dose TNFα induced much stronger and sustained activation of the Src-p190GAP pathway than non-cytotoxic dose TNFα, resulting in significant early-phage inactivation of RhoA (Fig. 4). Thus, cytotoxic dose TNFα-induced differential activation between Src-p190GAP and ERK-GEF-H1 pathways is responsible for significant early-inactivation of RhoA, resulting in substantial BAD release from the cytoskeleton to the cytosol for apoptosis. It is important to note that the crucial role that early-activation phase of RhoA plays in cytotoxic dose TNFα-induced BAD release from the cytoskeleton to the cytosol and subsequent translocation to mitochondria to trigger apoptosis is also supported by the observation that reconstituted RhoA(Q63L)^+^ MEFs were resistant to cytotoxic dose TNFα-induced BAD release from the cytoskeleton and mitochondrial translocation and apoptotic cell death (Fig. 4f–h). It is worthy to note that although RhoA was re-activated about half hour post-stimulation, non-phosphorylated BAD was still remained in the cytosol and at mitochondria (Figs. 3 and 4). It is likely that BAD is not immediately recruited to actin stress fibers even RhoA has been re-activated and that re-activation of RhoA may not lead to activation of the BAD kinases, which are involved in phosphorylation and thereby the removal of BAD from mitochondria.^32^ Future studies are needed to test these scenarios.

The fact that cytotoxic dose TNFα induced stronger activation of the Src-p190GAP pathway but comparable activation of the IKK-NF-κB pathway compared to non-cytotoxic dose TNFα suggests that activation of the IKK-NF-κB pathway is less sensitive to changes of TNFα concentration. This finding is consistent with a previous report, in which changes in activation of NF-κB was only a factor of four in response to a four order of magnitude change of TNFα concentration (from 0.01 ng/ml to 100 ng/ml;^76^). It is possible that both cytotoxic (80 ng/ml) and non-cytotoxic dose (5 ng/ml) TNFα may be near the “stimulation plateau” concentration in terms of activation of the IKK-NF-κB pathway, but not yet to do so in terms of activation of the Src-p190GAP pathway. Future studies are needed to exploit this scenario.

The pro-apoptotic activity of BAD is essential for TNFα cytotoxicity in vivo. Previously, it has been reported that loss of BAD suppressed but the IKK-non-phosphorylatable BAD mutant accelerated TNFα-induced mortality in mice in the presence of D-Galen, which is a protein synthesis inhibitor that can inhibit NF-κB activation in vivo.^14^ This finding is consistent with a previous report that BAD(3SA) mutant MEFs, in which the regulatory serines (Ser112, Ser136 and Ser155) have been replaced by non-phosphorylatable alanine, were more sensitive to TNFα-induced apoptosis.^44^ The notion that the pro-apoptotic activity of BAD is required for TNFα-induced apoptosis is further supported by the observations that phosphorylation of the regulatory serines depends on Ser26-phosphorylation by IKK in response to TNFα and *Bad*^3SA/3SA^ mice were also more sensitive to TNF-induced mortality.^14^ Consistently, our results show that loss of BAD reduced mortality rate and blocked tissue damage of liver and colon in polymicrobial infection-induced TNF-R1-dependent septic shock (Fig. 5). Polymicrobial infection-induced TNF-R1-dependent massive BAD release from the cytoskeleton to the cytosol, in which BAD became more than IKK could phosphorylate, resulting in mitochondrial translocation of the remaining non-phosphorylated BAD and subsequent apoptosis despite concurrent activation of the IKK signaling pathway (Fig. 6). Furthermore, BAD pro-apoptotic activity was essential for tissue damage of multiple organs and mortality (Fig. 7). Thus, the pro-apoptotic activity of BAD is essential for TNFα cytotoxicity in septic shock. However, these findings are contradictory to a recent report showing that there was no significant difference in TNFα-induced apoptosis in WT and *Bad*^−/−^ thymocytes, nor the mortality between WT and *Bad*^−/−^ mice in response to TNFα plus D-GalN.^77^ This apparent discrepancy is likely due to two reasons. First, thymocytes typically have modest basal level apoptosis, which can be significantly augmented by TNFα.^78, 79^ However, in Ottina’s study, the basal apoptosis of thymocytes was so high (~70%) without any explanations. Under this condition, TNFα did not further induce apoptosis of thymocytes even IKK was inhibited.^77^ Thus, it is not surprising that loss of BAD did not affect thymocyte apoptosis.^77^ Second, in Yan’s previous study the dose of TNFα was 15 μg/kg body weight (BW), which alone was unable to induce organ damage and mortality in mice.^14^ However, in Ottina’s study the dose of TNFα was 15 mg/kg BW, which is extremely high and most likely results in indiscriminately killing of both WT and *Bad*-deficient mice.^77^ Taken together, BAD is involved in TNFα cytotoxicity under physiological and pathological conditions.

Blocking BAD-mediated TNFα cytotoxicity may provide a novel strategy for prevention and treatment of septic shock. Although it is known that systemic inflammation is responsible for multiple organs failure and mortality in septic shock, the anti-inflammation therapies including anti-TNFα therapy are largely unsuccessful, partly due to losing the beneficial inflammatory response-mediated host defense against bacteria.^2–4^ Our results show that loss of Bad inhibited tissue damage of liver and colon associated with massive apoptosis in septic shock (Fig. 5), consistent with previous reports that TNFα-induced apoptosis was associated with tissue damage of multiple organs in septic shock ^25–28^ and reduced the mortality rate (Fig. 5a). Specific blockade of substantial BAD release from the cytoskeleton to the cytosol may prevent or at least reduce TNFα cytotoxicity in inflammatory and infectious diseases. Given the importance of TNFα cytotoxicity in the pathologies of many inflammatory and infectious diseases,^67–69^ our finding is likely to have broad implications in prevention and treatment of inflammatory and infectious diseases.

## Materials and methods

### Mice

*Bad*^−/−^ mice were a generous gift of Dr. Nike N. Danial (Dana-Farber Cancer Institute) and have been backcrossed into the C57BL/6J genetic background for at least 14 generations and validated by genome scanning to be 99.9% congenic with C57BL/6J.^80^ Heterozygous *Bad* knockout mice were further bred to generate age-matched WT littermate and knockout experimental mice. *Tnf-*R1^−/−^ mice in C57BL/6J genetic background were purchased from Jackson laboratory, and then bred with C57BL/6J WT mice for heterozygous mice. Heterozygous *Tnf-*R1 knockout mice were further bred to generate age matched WT littermate and knockout experimental mice. For the rescue experiments, *Bad*^−/−^ mice were injected through tail vein with recombinant adenoviral vector encoding *Bad*(WT)^+^, *Bad*(S26D)^+^, *Bad*(S26A)^+^, and the control *Bad*(LacZ)^+^ (a total dose of 4 × 10^9^ infectious units per ml [IFUs] in a volume of 100 μl per animal) to generate *Bad*(WT)^+^, *Bad*(S26D)^+^, *Bad*(S26A)^+^ and *Bad*(LacZ)^+^ mice respectively.^14^ Gender and age-matched (male, 6–8 weeks old) *Bad*^−/−^ mice and their WT littermate, and *Tnf*-R1^−/−^ mice and their WT littermate, as well as *Bad*(WT)^+^, *Bad*(S26A)^+^, *Bad*(S26D)^+^ and the control *Bad*(LacZ)^+^ mice were used. For the polymicrobial infection model, the CLP procedure was performed.^50^ The severity of CLP was adjusted to a high-grade sepsis by ligating the cecum three fourth from the tip of cecum and single through-and-through puncture with a 21 G needle between the ligation and the tip of the cecum in a mesenteric-to-antimesenteric direction.^50^ For the mock CLP procedure (Sham), mice underwent the same abdominal surgery as CLP procedure, except the cecal ligation and puncture. The LPS model was performed by intraperitoneally (i.p.) injection of lethal dose LPS (*E. coli* serotype 0111:B4; Sigma; 35 mg/kg body weight), respectively, as indicated in figure legends. Mice were sacrificed for analysis of BAD subcellular localizations, blood bacteria burden, production of pro-inflammatory and anti-inflammatory cytokines, serology and liver and colon histology. Mortality rate was recorded for up to 120 h for CLP model, 50–60 h for LPS model and dying mice were pre-moved. The statistics analysis was performed by the log rank (Mantel-Cox) test. The experimental mice were allocated based on the same gender, similar age and body weight of the littermate without selection. The mice treatment and sample collection were blind, as it was done based on the ear-tag number without knowing the group information.

To analyze tissue injury, liver lobes and colon were excised and fixed in 4% paraformaldehyde for 12 h. The tissues were sliced to 5 μm thickness. Hematoxylin and eosin (H&E) staining was performed at the University of Chicago Human Tissue Resource Center (HTRC). In situ cell death was analyzed by TUNEL staining (TUNEL Apoptosis Detection kit, EMD Millipore), according to the manufacturer’s protocol.

Blood samples were collected from tail vein of animals at different time points as indicated in the figure legends. Serum from the blood samples was collected after centrifugation. ALT, AST, BUN and LDH levels were measured with the BioAssay systems kits (Bioassay Systems), according to the manufacturer’s protocol.

To analyze the bacterial load in circulation and target organs of severe sepsis, blood and tissue samples were collected at different time points as indicated in the figure legends. The same amount of tissue homogenate or blood plasma was plated on agar plates (TSA II, Trypticase Soy Agar with 10% Sheep Blood; Becton Dickinson) in 5 serial 10-fold dilutions. Following overnight incubation, bacterial colonies were performed with Gram staining and enumerated. At least 8 plates for each sample were calculated.

All mice were maintained in specific pathogen-free facility and housed on a 12:12-h light/dark photoperiod at an ambient temperature of 22 ± 2^o^C. All animal experiments were conducted in accordance with the protocols approved by the Institutional Animal Care and Use Committee of the University of Chicago.

### Cells

WT, *Bad*^−/−^ and *Ikk*β^−/−^ fibroblasts have been described previously.^11, 14^ *Tnf-*R1^−/−^ fibroblasts were gift from Dr. Zhenggang Liu at NCI and *Rho*A^f/f^ primary fibroblasts were a gift from Dr. Yi Zheng at University of Cincinnati.^81^ *RhoA*(WT)^+^ and *RhoA*(Q63L)^+^ MEFs were prepared by infection of RhoA^f/f^ primary fibroblasts with Ad/cre (10 multiplicity of infection, moi), followed by transfection with expression vector pcDNA3 (2 μg) encoding WT RhoA or the RhoA(Q63L) mutant, in which glutamine residue 63 has been replaced by leucine. Cells were grown in Dulbecco’s modified Eagle’s medium supplemented with 10% fetal bovine serum, 2 mM glutamine, 100 U/ml penicillin, and 100 μg/ml streptomycin.

For isolation and culture of primary hepatocytes, mice were perfused with perfusion medium (Invitrogen) and digested with 100 U/ml collagenase. The collected primary hepatocytes were cultured in DMEM/F12 medium containing 10% FBS, 1× Insulin-Transferrin-Selenium and 10 μg/ml EGF. For preparing primary murine macrophages, the whole bone marrow was harvested from femur and tibia bones. After removing the mixed red blood cells by ACK lysis buffer, cells were re-suspended in BMDM conditional medium containing 20% FBS, 14.3 μM β-mercaptoethanol and 20 ng/ml M-CSF. For isolation and culture of primary thymocytes, the murine thymuses were extracted aseptically and collected in RPMI 1640 medium. Single cell suspensions of the thymuses were prepared by press the organs through 70 μm nylon cell strainer and cultured in RPMI 1640 medium with 10% FBS. For isolation of primary splenocytes, spleens were extracted from mice and gently pressed through 70 μm nylon cell strainer. The mixed red blood cells were removed by ACK lysis buffer. After centrifugation at 800 × g for 3 min, the pellet of splenocytes was re-suspended in Dulbecco’s modified Eagle’s medium supplemented with 10% fetal bovine serum.

### Reagents

Antibodies against BAD, TNF-R1, cofilin, ERK, IκBα, p190GAP, RhoA, Src, phospho-ERK, phospho-IκBα, phospho-IKKβ, phospho-Tyr416-Src, phospho-Tyrosine were from Cell signaling. Anti-phospho-BAD(S26) antibody was from Abiocode Inc. Antibodies against BCL-x_L_, COX-2, desmoplakin, GAPDH, vimentin were from Santa Cruz. Antibody against α-Tubulin was from Abcam. Antibodies against GEF-H1 and MAP1β were from Thermo. Antibody against IKKβ was from EMD Millipore. Antibody against β-actin was from Sigma. Antibody against MLKL was from Biorbyt. Purified GST and GST-BAD fusion proteins were described previously.^14^ Purified GST-Cofilin fusion proteins were purchased from BPS Bioscience. Purified G-actin monomer proteins were purchased from Cytoskeleton. z-VAD, Cycloheximide, Hoechst and Cytochalasin D were from Sigma. Jasplakinolide was from Biovision. Sphingosine-1-phosphate (S1P) was from Cayman chemical. Latrunculin B was from Enzo Life Science. Alexia fluorescein tagged second antibody and MitoTracker were from Invitrogen. TNFα (murine) was from R&D, GeneScript and PeproTech. The fractions of the TNFα preparation used in this study was customized prepared by R&D. The total 21 different fractions in the final purification step was collected individually, an aliquot of which was also pooled together (Fig. 1f; Supplementary information, Figure S1g and Table S3).

The sequences of siRNA are siBAD: AGCUCCUGUUUGGAGUUUCAAA, and siCtrl: GGAGCGCACCAUCUUCUUC, were synthesized from IDT. siSrc SMART pool were from GE Dharmacon. sip190GAP was from Santa Cruz.

### MS/MS spectrometry analysis of the purity of TNFα and potential contaminated small chemical compounds in TNFα

Filter-aided sample preparation (FASP) was applied to extract and digest TNFα protein. The digested peptides were then dried and reconstituted in 0.1% FA. One μg protein was injected into Thermo Fisher Orbitrap Velos Pro coupled with Agilent NanoLC system (Agilent, Santa Clara, CA) over a 30-min gradient. RAW files were converted into.mgf files using MSConvert (from ProteoWizard). Database search was carried out using Mascot server (from Matrix Science) against UNIPROT mouse and *E. coli* database. The Mascot search results against mouse were shown in Supplementary information, Table S1. TNFα was the top hit with 55% TNFα sequence coverage, covering from AA82 to AA 218. The covered sequence showed no major modifications. No positive hits came up against *E. coli* search, indicating that host-cell proteins are below limit of detection.

To detect potential contaminated small chemical compounds in TNFα, 150 μl acetonitrile was added to 20 μl TNFα solution. After centrifugation, the supernatant was collected and dried. The supernatant was reconstituted by adding 10 μl of 50:50 acetonitrile and distilled deionized water. The sample was transferred into sample vial for LC-MS/MS analysis. Two μl of the sample was injected into the LCMS IT-TOF. In both positive and negative mode, the precursor ions observed in the sample during a full MS scan (in the mass range of 100–2,000 *m*/*z*) were the same as those observed in the solvent blank (standard PBS). The mass spectrum was shown in Supplementary information, Table S2. The MS spectrometry analyses were performed by University of Illinois at Chicago, UICentre.

### Flow cytometric assay and quantitative real-time PCR

For flow cytometric analysis, cells were harvested by Trypsin digestion after different treatments, as indicated in figure legends. Annexin V and Propidium iodide (PI) staining was performed with FITC Annexin V apoptosis Detection kit (BD Pharmingen). Apoptotic cells were analyzed by FACS canto flow cytometer (BD Biosciences). For quantitative real-time PCR analysis, total RNA was extracted by Trizol reagent (Invitrogen) from cells, as indicated in figure legends. Total RNA (1 μg) was used for cDNA synthesis. SYBR Green real-time PCR master Mix (Invitrogen) and Taqman PCR program was used for real-time PCR. PCR primers were synthesized from IDT. GAPDH was used as internal control. The sequences of primers were as follows:

mTNFα: sense, 5′-CACAGAAAGCATGATCCGCGACGT-3′; antisense, 5′-CGGCAGAGAGGAGGTTGACTTTCT-3′

IFNγ: sense, 5′-TCAAGTGGCATAGATGTGGAAGAA-3′; antisense, 5′-TGGCTCTGCAGGATTTTCATG-3′

IL-1β: sense, 5′-CCAGCTTCAAATCTCACAGCA-3′; antisense, 5′-CTTCTTTGGGTATTGCTTGGGATC-3′

IL-10: sense, 5′-GGTTGCCAAGCCTTATCGGA-3′; antisense, 5′-ACCTGCTCCACTGCCTTGCT-3′

IL-6: sense, 5′-TCCAGTTGCCTTCTTGGGAC-3′; antisense, 5′-GTACTCCAGAAGACCAGAGG-3′

IκBα: sense, 5′-TGAAGGACGAGGAGTACGAGC-3′; antisense, 5′-TTCGTGGATGATTGCCAAGTG-3′

cIAP2: sense, 5′-ACGCAGCAATCGTGCATTTTG-3′; antisense, 5′-CCTATAACGAGGTCACTGACGG-3′

GAPDH: sense, 5′-AACGACCCCTTCATTGAC-3′; antisense, 5′-TCCACGACATACTCAGCAC-3′

### Cytokine assay, immunoprecipitation, caspase 3 assay, G-LISA assay, active GEF-H1 assay, tissue and cell fractionations, and subcellular localization analysis and calculation

Cytokines in circulation were measured with Cytometric Bead Array (CBA) Mouse Inflammation kit (BD biosciences), according to the manufacturer’s protocol.

Immunoprecipitation was performed as previously described.^14^ Caspase 3 assay was performed with Casp-3 cellular activity assay kit using Casp-3 substrate Ac-DEVD-pNA (Calbiochem). Active RhoA and active Cdc42 assays were performed with G-LISA assay biochemical kit (Cytoskeleton, Inc.). Active GEF-H1 assays were performed by using purified GST-RhoA(G17A) to pull down the active GEF-H1 from cell extracts and analyzed by immunoblotting. Cytosol and mitochondria fractionation was carried out by using the cytosol/mitochondria fractionation kit (Biovision), according to the manufacturer’s protocol. Note, using this method, the cytosol fraction also contains the cytoskeleton. On the other hand, cytosol and cytoskeleton fractionation was carried out by using the subcellular protein fractionation kit for culture cells (Thermo), according to the manufacturer’s protocol with 3 mM ATP in the F-actin stabilization buffer. Note, using this method, the cytosol fraction also contains subcellular organelles including mitochondria. Cytoskeleton precipitation and G-actin/F-actin assay were performed by the G-actin/F-actin in vivo assay biochem kit (Cytoskeleton, Inc.).

Subcellular localization of BAD proteins was determined by immunoblotting with anti-BAD antibody and quantitated by the ImageJ program and/or IRDye with Li-cor Odyssey imaging system. At each time point, the sum of BAD proteins in different fractions, either cytosol (containing the cytoskeleton) vs. mitochondria, or cytoskeleton vs. cytosol (containing mitochondria), was calculated as 100%. To calculate the percentage of IKK-phosphorylated BAD in total cytoplasmic BAD, Ser26-phosphorylated BAD proteins were immunoprecipitated with anti-Ser26 antibody and immunoblotted with anti-BAD antibody and quantitated by the ImageJ program and/or IRDye with Odyssey imaging system. BAD proteins from the same amount of total cell extracts were detected with anti-BAD antibody and calculated as 100%.

### Actin filament binding assay and G-actin monomer GST protein pulldown

The actin filament binding assay was performed by using the actin filament binding protein spin-down assay kit (Cytoskeleton Inc.), according to the manufacturer’s protocol. Briefly, purified G-actin monomers (5 μg) were polymerized by incubation at room temperature for 1 h and then purified GST-BAD proteins, GST-Cofilin (positive control) or GST (negative control) (2 μg each) were added separately to the reaction mixture to incubate at room temperature for another 30 min. F-actin filament-associated GST-BAD or GST-Cofilin proteins were obtained by ultracentrifugation for 20 min and subjected to immunoblotting assay with anti-GST antibody, as indicated in Supplementary Information, Figure S3c (Filament pellet panels). The possible interaction between G-actin monomers and BAD was detected by GST protein pulldown assay. Briefly, purified G-actin monomers were incubated with purified GST-BAD, GST (negative control), or GST-Cofilin (positive control) separately in vitro. GST-fusion protein-associated G-actin was precipitated by Glutathione Sepharose beads and analyzed by immunoblotting assay with anti-actin antibody, as indicated in Supplementary Information, Figure S3c (GSH pulldown panels).

### Immunofluorescence staining, Ground State Depletion (GSD) microscopy, proximity ligation assay

Immunofluorescence staining was performed as previously described.^14^ Images were captured using Leica SP5 Tandem Scanner Spectral 2-Photon Confocal equipped with Leica LAS-AF software. The fluorescence intensity was quantitated by single line scan analysis using the ImageJ program in six random fields.

GSD imaging was visualized by Leica GSD/TIRFM ground state depletion super-resolution microscope using 160 × /1.43 oil HCX PL APO objective and Andor-DU897_BV-7849 camera. Proximity ligation assay was performed with Duolink in situ fluorescent detection reagents (Sigma). Images were taken by Leica SP5 Tandem Scanner Spectral 2-Photon Confocal, and quantitated by the ImageJ program. Eight different fields of each sample were randomly chosen for analysis.

### Statistical analysis

Cell death (apoptosis or necroptosis) assays, Casp-3 assays, quantitative real-time PCR analysis, G-LISA assays and Src kinase assay, bacterial load, cytokine detection and serology measurement between two groups were expressed as means ± s.d. Statistical analysis was conducted using two-tailed unpaired Student’s *t* test. Mouse survival curves were constructed using the Kaplan–Meier product limit estimator and compared using the log rank (Mantel-Cox) test. *P* < 0.05 was considered to be significant in all experiments.

## Electronic supplementary material

Supplementary information

Supplementary information, Movie S1

Supplementary information, Movie S2
