# Supplementary material for: The BH3-only protein BAD mediates TNFα cytotoxicity despite concurrent activation of IKK and NF-κB in septic shock
Source: Cell Res. 2018 May 24;28(7):701–18. doi: 10.1038/s41422-018-0041-7 (PMC6028455; doi:10.1038/s41422-018-0041-7)
Supplement: Supplementary file 1 — Supplementary information [file 41422_2018_41_MOESM1_ESM.pdf]

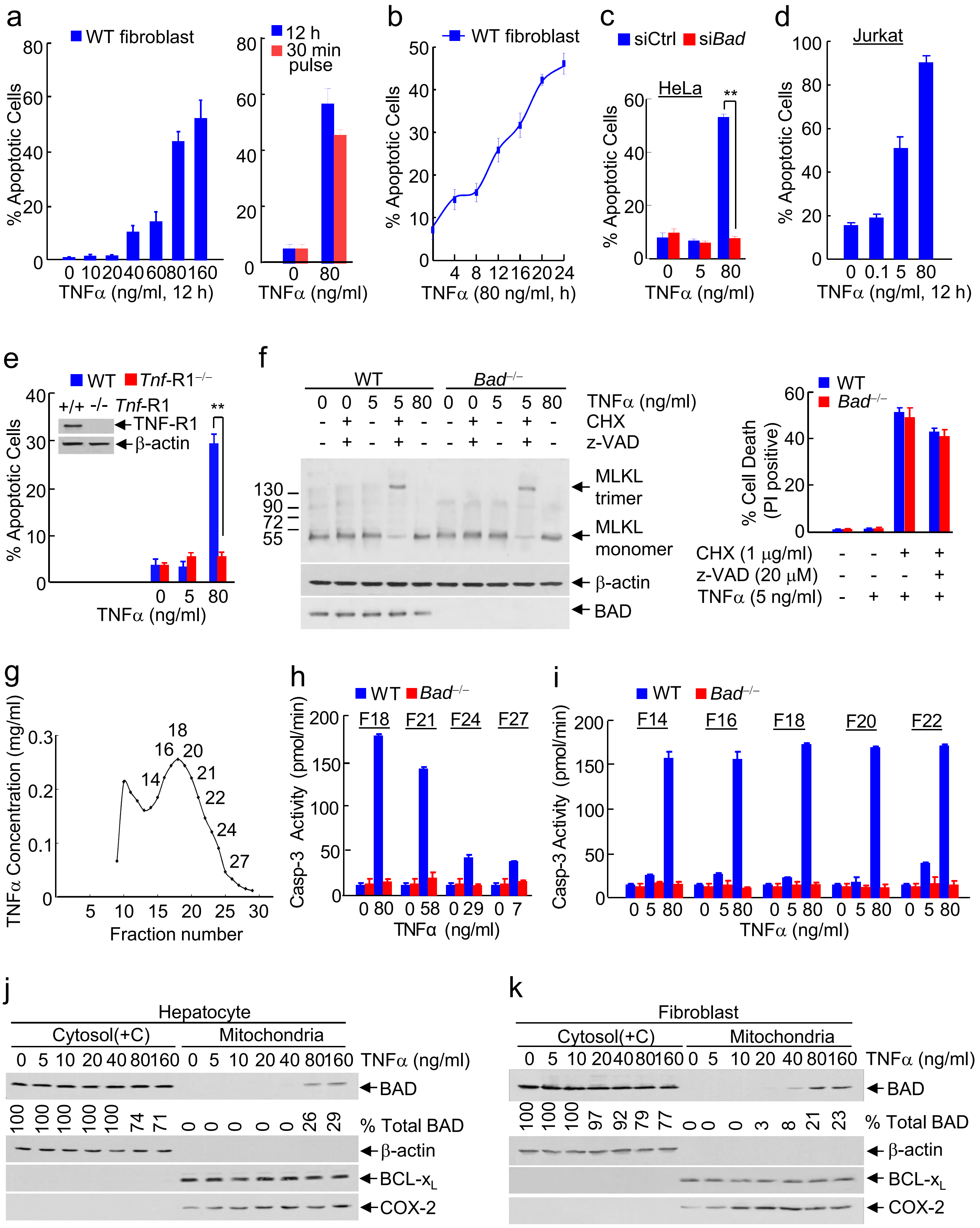
a
WT fibroblast
% Apoptotic Cells
TNFα (ng/ml, 12 h)
12 h
30 min pulse
TNFα (ng/ml)
b
WT fibroblast
% Apoptotic Cells
TNFα (80 ng/ml, h)
c
siCtrl
siBad
HeLa
% Apoptotic Cells
TNFα (ng/ml)
d
Jurkat
% Apoptotic Cells
TNFα (ng/ml, 12 h)
e
WT
Tnf-R1−/−
+/+ -/- Tnf-R1
TNF-R1
β-actin
% Apoptotic Cells
TNFα (ng/ml)
f
WT
Bad−/−
TNFα (ng/ml)
CHX
z-VAD
MLKL trimer
MLKL monomer
β-actin
BAD
% Cell Death (PI positive)
CHX (1 μg/ml)
z-VAD (20 μM)
TNFα (5 ng/ml)
g
TNFα Concentration (mg/ml)
Fraction number
h
WT
Bad−/−
F18 F21 F24 F27
Casp-3 Activity (pmol/min)
TNFα (ng/ml)
i
WT
Bad−/−
F14 F16 F18 F20 F22
Casp-3 Activity (pmol/min)
TNFα (ng/ml)
j
Hepatocyte
Cytosol(+C)
Mitochondria
TNFα (ng/ml)
BAD
% Total BAD
β-actin
BCL-xL
COX-2
k
Fibroblast
Cytosol(+C)
Mitochondria
TNFα (ng/ml)
BAD
% Total BAD
β-actin
BCL-xL
COX-2

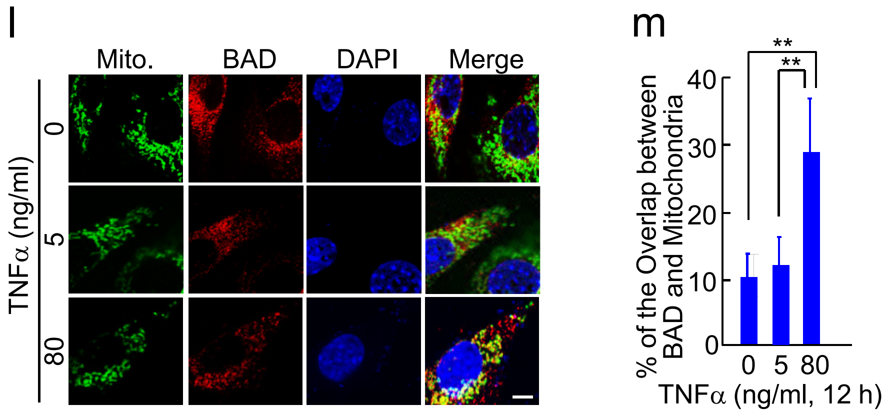

Supplementary Information, Figure S1 TNFα induces apoptosis, but not necroptosis, and BAD mitochondrial translocation in a dose-dependent and time-dependent manner. (a-b) Immortalized fibroblasts were treated without or with various doses of TNFα for 12 h (**a, Left panel**) or treated with cytotoxic dose TNFα (80 ng/ml) for 30 min and then replaced with fresh medium containing no TNFα for 12 h (**a, Right panel**), or treated with cytotoxic dose (80 ng/ml) TNFα for various times **(b)**, as indicated. Apoptotic cells was determined by Annexin V/Propidium iodide (PI) staining and flow cytometric analysis. **(c-d)** HeLa cells (**c**) transfected with siBad or the control scramble siRNA, or Jurkat cells **(d)** were treated without or with various doses of TNFα for 12 h, as indicated. Apoptotic cell death were detected as described in **(a)**. **(e)** WT and *Tnf*-R1$^{-/-}$ fibroblasts were treated without or with non-cytotoxic or cytotoxic dose TNFα for 12 h, as indicated. Apoptotic cell death were detected as described in **(a)**. **(f)** WT and *Bad*$^{-/-}$ fibroblasts were treated without or with non-cytotoxic (5 ng/ml) in the presence of CHX (1 μg/ml) and pan-caspase inhibitor z-VAD-FMK (20 μM) or cytotoxic dose (80 ng/ml) TNFα for 6 h (MLKL Trimerization) or 12 h (Cell death assay), as indicated. MLKL trimerization was detected by non-reducing gel (SDS-PAGE without β-ME and DTT) and analyzed by immunoblotting of MLKL, BAD, and β-actin using corresponding antibodies (**Left panel**). Cell death was determined by PI staining and flow cytometric analysis (**Right panel**). **(g-i)** Different fractions (F, fraction; F14, Fraction number 14 and etc.) of the TNFα preparation (R&D) **(g)** were used to treat WT and *Bad*$^{-/-}$ immortalized fibroblasts, as indicated, and caspase-3 activity were detected (**h-i**). The concentration of TNFα in different fractions was shown in Table S3. **(j-k)** Primary hepatocytes **(j)** or immortalized fibroblasts **(k)** were treated without or with various doses of TNFα for 4 h, as indicated. BAD mitochondrial translocation was determined by cytosol [containing cytoskeleton, Cytosol(+C)] and mitochondria fractionation (see "Materials and Methods" for details), followed by immunoblotting with corresponding antibodies, as indicated. **(l-m)** WT fibroblasts were treated without or with non-cytotoxic dose or cytotoxic dose TNFα for 12 h and the co-localization of BAD with mitochondria was analyzed by immunofluorescence staining using anti-BAD antibody, MitoTracker and DAPI (4',6-diamidino-2-phynylindole). Images were taken with confocal microscopy. Scale bar, 5 μm **(l)**. Co-localization of BAD with mitochondria in **(l)** was quantitated by the ImageJ program in six random fields and determined by Pearson's correlation coefficient **(m)**. Data in **(a-f, h-i and m)** are means ±s.d. **, $P < 0.01$, as analyzed by two-tailed unpaired Student's *t* test. All data represent two to three individual experiments with similar results.

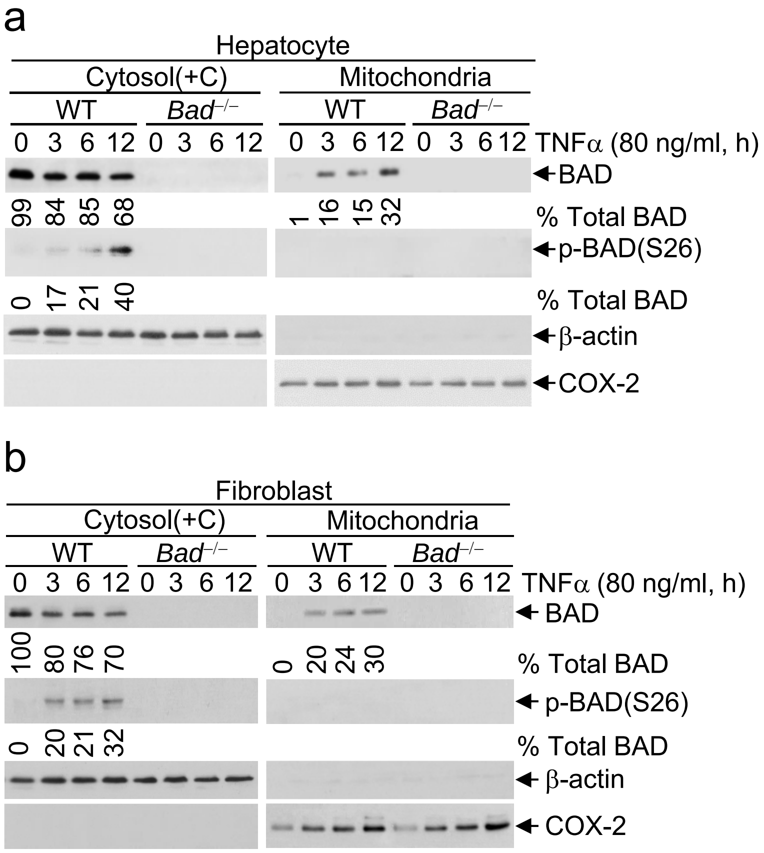

Supplementary Information, Figure S2 Only a fraction of cytosolic BAD is phosphorylated in response to cytotoxic dose TNFα and the rest non-phosphorylated BAD translocates to mitochondria. Primary hepatocytes **(a)** and immortalized fibroblasts **(b)** were treated without or with cytotoxic dose TNFα for various times, as indicated. BAD phosphorylation by IKK and BAD mitochondrial translocation were determined by cytosol [containing the cytoskeleton, Cytosol(+C)] and mitochondria fractionation (see “Materials and Methods" for details) and immunoblotting. The percentage of IKK-phosphorylated BAD in total cytoplasmic BAD protein was determined as described in **(Figure 1f, Supplementary information, S9t and S9u)**. All data represent two to three individual experiments with similar results.

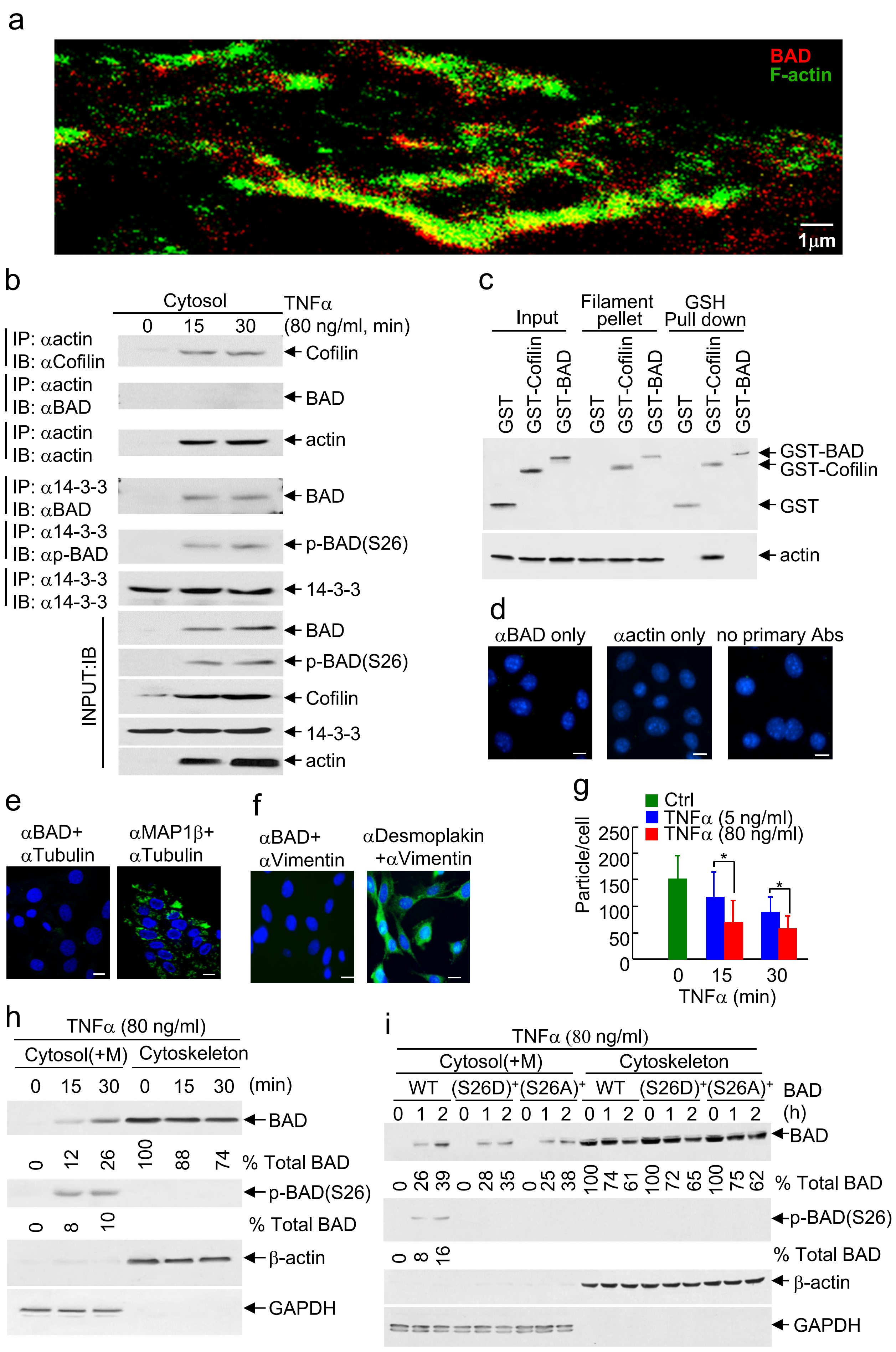
a
BAD
F-actin
1μm
b
Cytosol
TNFα (80 ng/ml, min)
0 15 30
IP: αactin IB: αCofilin
Cofilin
IP: αactin IB: αBAD
BAD
IP: αactin IB: αactin
actin
IP: α14-3-3 IB: αBAD
BAD
IP: α14-3-3 IB: αp-BAD
p-BAD(S26)
IP: α14-3-3 IB: α14-3-3
14-3-3
INPUT:IB
BAD
p-BAD(S26)
Cofilin
14-3-3
actin
c
Input
Filament pellet
GSH Pull down
GST GST-Cofilin GST-BAD GST GST-Cofilin GST-BAD GST GST-Cofilin GST-BAD
GST-BAD
GST-Cofilin
GST
actin
d
αBAD only
αactin only
no primary Abs
e
αBAD+ αTubulin
αMAP1β+ αTubulin
f
αBAD+ αVimentin
αDesmoplakin +αVimentin
g
Ctrl
TNFα (5 ng/ml)
TNFα (80 ng/ml)
Particle/cell
250 200 150 100 50 0
0 15 30
TNFα (min)
h
TNFα (80 ng/ml)
Cytosol(+M)
Cytoskeleton
0 15 30 0 15 30 (min)
BAD
0 12 26 100 88 74 % Total BAD
p-BAD(S26)
0 8 10 % Total BAD
β-actin
GAPDH
i
TNFα (80 ng/ml)
Cytosol(+M)
Cytoskeleton
WT (S26D)+ (S26A)+ WT (S26D)+ (S26A)+ BAD
0 1 2 0 1 2 0 1 2 0 1 2 0 1 2 0 1 2 (h)
BAD
0 26 39 0 28 35 0 25 38 100 74 61 100 72 65 100 75 62 % Total BAD
p-BAD(S26)
0 8 16 % Total BAD
β-actin
GAPDH

Supplementary Information, Figure S3 BAD directly interacts with F-actin in actin stress fibers at the cytoskeleton. (a) WT fibroblasts were analyzed by double immunofluorescence staining with anti-BAD antibody (red) and phalloidin (actin filament marker; green), and visualized with super-resolution microscopy based on Ground State Depletion program. Scale bar, 1 μm. **(b)** WT fibroblasts were treated without or with cytotoxic dose TNFα for various times, as indicated. The cytosol fractions were separated from the cytoskeleton fractions by ultracentrifugation (see "Materials and Methods" for details). Potential protein-protein association between G-actin and BAD in the cytosol was analyzed by immunoprecipitation with anti-actin antibody, followed by immunoblotting with anti-BAD antibody. The associations between G-actin and Cofilin and 14-3-3 with phosphorylated BAD were used as positive controls. IKK-phosphorylation of BAD, expression levels of BAD, Cofilin, G-actin and 14-3-3 were determined by immunoblotting with corresponding antibodies (Input). **(c)** Purified G-actin monomers were incubated with purified GST, GST-Cofilin or GST-BAD protein separately *in vitro*. An aliquot of the mixture was subjected to ultracentrifugation to detect the interaction between GST-fusion proteins and F-actin in actin filaments. In parallel, another aliquot of the mixture was used in GST pulldown assay to detect the interaction between GST-fusion proteins and G-actin monomers. GST-fusion proteins and actin were detected by immunoblotting with anti-GST and anti-actin antibodies, respectively. A third aliquot of the mixture was used as Input. **(d-f)** Negative controls **(d)** and positive controls **(e-f)** for proximity ligation assay **(Figure 3b).** WT fibroblasts were stained with anti-BAD antibody only, anti-β-actin antibody only, and no primary antibodies **(d)**, or anti-MAP1β and anti-Tubulin **(e)**, anti-Desmoplakin and anti-Vimentin **(f)** instead, as indicated. Nuclei were detected by DAPI. Scale bar, 5 μm. **(g)** Quantitation of co-localization of BAD with actin by proximity ligation assay **(Figure 3b)** with the ImageJ program in eight different fields. Data are means ±s.d. *, $P < 0.05$, as analyzed by two-tailed unpaired Student's *t* test. **(h)** WT fibroblasts were treated without or with cytotoxic dose TNFα for various times, as indicated, followed by the cytoskeleton fractionation (see "Materials and Methods" for details). The percentage of IKK-phosphorylated BAD in total cytoplasmic BAD protein was determined as described (**Figure 1f) (Supplementary information, Figure S9v)**. **(i)** Bad-deficient fibroblasts were transfected with WT Bad, Bad(S26D) or Bad(S26A) mutant, followed by treatment without or with 80 ng/ml TNFα for various times, as indicated. Subcellular localizations of BAD and IKK-phosphorylated BAD were analyzed and quantitated as described in **(h) (Supplementary information, Figure S9w)**. All data represent two to three individual experiments with similar results.

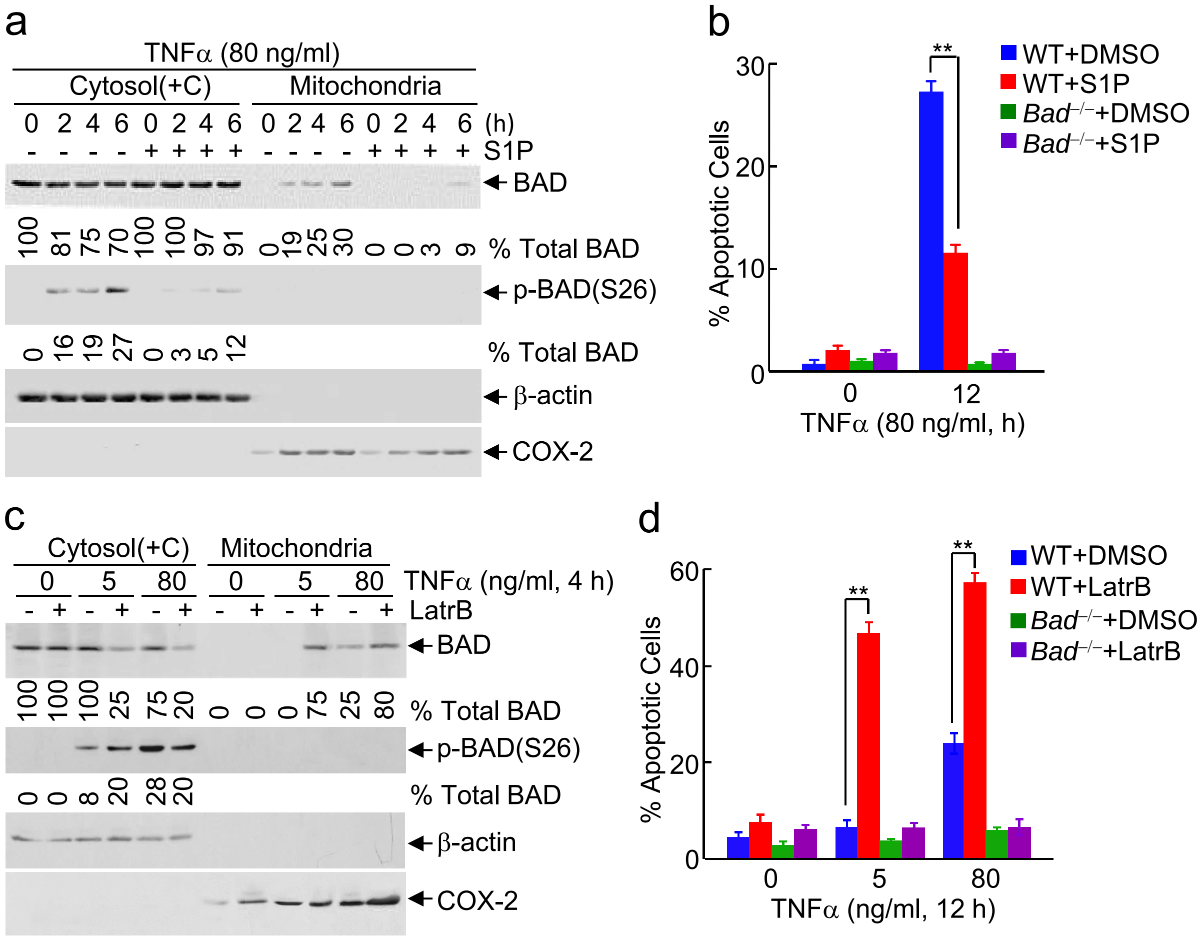

Supplementary Information, Figure S4 The ability of TNFα to induce apoptosis is regulated by actin stress fiber polymerization. WT and *Bad*$^{-/-}$ fibroblasts were pretreated without or with 5 μM Sphingosine-1-phosphate (S1P) **(a-b)**, 2.5 μg/ml Latrunculin B **(c-d)** or DMSO (control) for 1 h, followed by stimulation without or with non-cytotoxic or cytotoxic dose TNFα for various times, as indicated. BAD phosphorylation and BAD mitochondrial translocation were determined by cytosol [containing cytoskeleton, Cytoso(+C)] and mitochondria fractionation (see “Materials and Methods" for details) and immunoblotting **(a, c)**. The percentage of IKK-phosphorylated BAD in total cytoplasmic BAD protein **(a, c)** was determined, as described in **(Figure 1f and Supplementary Information, Figure S9x-S9y)**. Apoptotic cells were determined as described in (**Figure 1a**) and present as means ±s.d. **, $P < 0.01$, as analyzed by two-tailed unpaired Student’s *t* test **(b, d)**. All data represent two to three individual experiments with similar results.

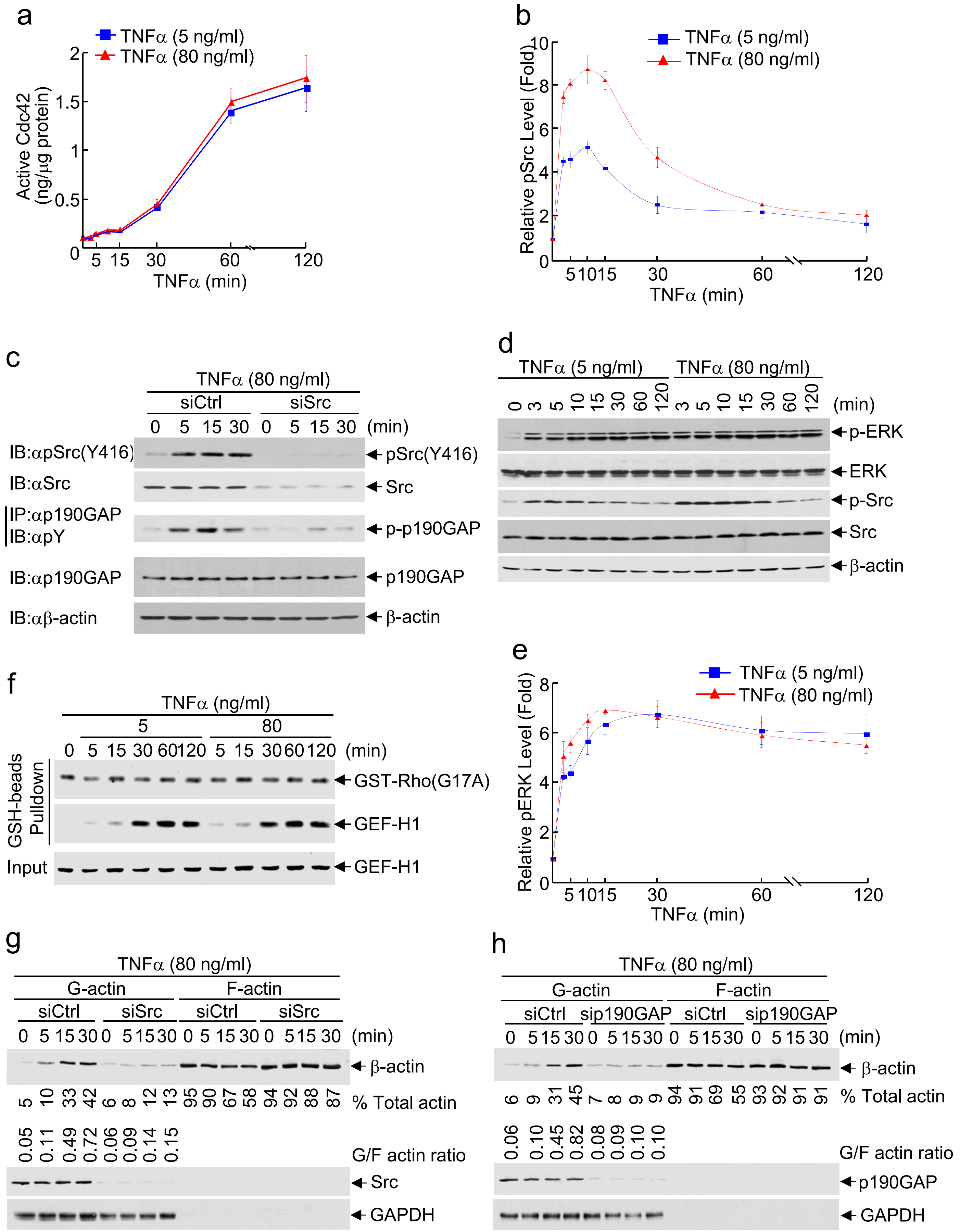
a
TNFα (5 ng/ml)
TNFα (80 ng/ml)
Active Cdc42 (ng/μg protein)
0
0.5
1
1.5
2
5 15 30 60 120
TNFα (min)
b
TNFα (5 ng/ml)
TNFα (80 ng/ml)
Relative pSrc Level (Fold)
0
2
4
6
8
10
5 10 15 30 60 120
TNFα (min)
c
TNFα (80 ng/ml)
siCtrl
siSrc
0 5 15 30 0 5 15 30 (min)
IB:αpSrc(Y416)
pSrc(Y416)
IB:αSrc
Src
IP:αp190GAP
IB:αpY
p-p190GAP
IB:αp190GAP
p190GAP
IB:αβ-actin
β-actin
d
TNFα (5 ng/ml)
TNFα (80 ng/ml)
0 3 5 10 15 30 60 120 3 5 10 15 30 60 120 (min)
p-ERK
ERK
p-Src
Src
β-actin
e
TNFα (5 ng/ml)
TNFα (80 ng/ml)
Relative pERK Level (Fold)
0
2
4
6
8
5 10 15 30 60 120
TNFα (min)
f
TNFα (ng/ml)
5
80
0 5 15 30 60 120 5 15 30 60 120 (min)
GSH-beads Pulldown
GST-Rho(G17A)
GEF-H1
Input
GEF-H1
g
TNFα (80 ng/ml)
G-actin
F-actin
siCtrl
siSrc
siCtrl
siSrc
0 5 15 30 0 5 15 30 0 5 15 30 0 5 15 30 (min)
β-actin
5 10 33 42 6 8 12 13 95 90 67 58 94 92 88 87 % Total actin
0.05 0.11 0.49 0.72 0.06 0.09 0.14 0.15
G/F actin ratio
Src
GAPDH
h
TNFα (80 ng/ml)
G-actin
F-actin
siCtrl
sip190GAP
siCtrl
sip190GAP
0 5 15 30 0 5 15 30 0 5 15 30 0 5 15 30 (min)
β-actin
6 9 31 45 7 8 9 9 94 91 69 55 93 92 91 91 % Total actin
0.06 0.10 0.45 0.82 0.08 0.09 0.10 0.10
G/F actin ratio
p190GAP
GAPDH

Supplementary Information, Figure 5 Cytotoxic dose TNFα induces massive depolymerization of actin stress fibers through Src-p190GAP-meidated inactivation of RhoA. **(a)** Cytotoxic and non-cytotoxic dose TNFα induced similar level of Cdc 42 activation, as measured by the G-LISA Cdc42 activation assay. Data are means ±s.d. **(b-e)** WT fibroblasts were treated without or with non-cytotoxic or cytotoxic dose TNFα for various times as indicated. Phospho-Src, phospho-ERK. Expression levels of Src, ERK and β-actin were determined by immunoblotting **(c-d)** and the fold of phosphorylation of Src and ERK were quantitatively analyzed by ImageJ program and presented as means ±s.d. **(b, e)**. Phosphorylated p190GAP was detected by immunoprecipitation with anti-p190GAP antibody and immunoblotting with anti-phosphor-Tyrosine (αpY) antibody **(c)**. **(f)** Active GEF-H1 was detected (see "Materials and Methods" for details). Expression level of GEF-H1 and the input GST-Rho(G17A) were detected with immunoblotting. **(g-h)** WT fibroblasts were transfected with scramble siRNA (siCtrl) **(g-h)**, siSrc **(g)** or sip190GAP **(h)**, and treated without or with cytotoxic dose TNFα for various times, as indicated. G-actin and F-actin were detected by fractionation and immunoblotting, and quantitated by the ImageJ program. Expressing level of Src **(g)** and p190GAP **(h)** was detected with corresponding antibodies, respectively. All data represent two to three individual experiments with similar results.

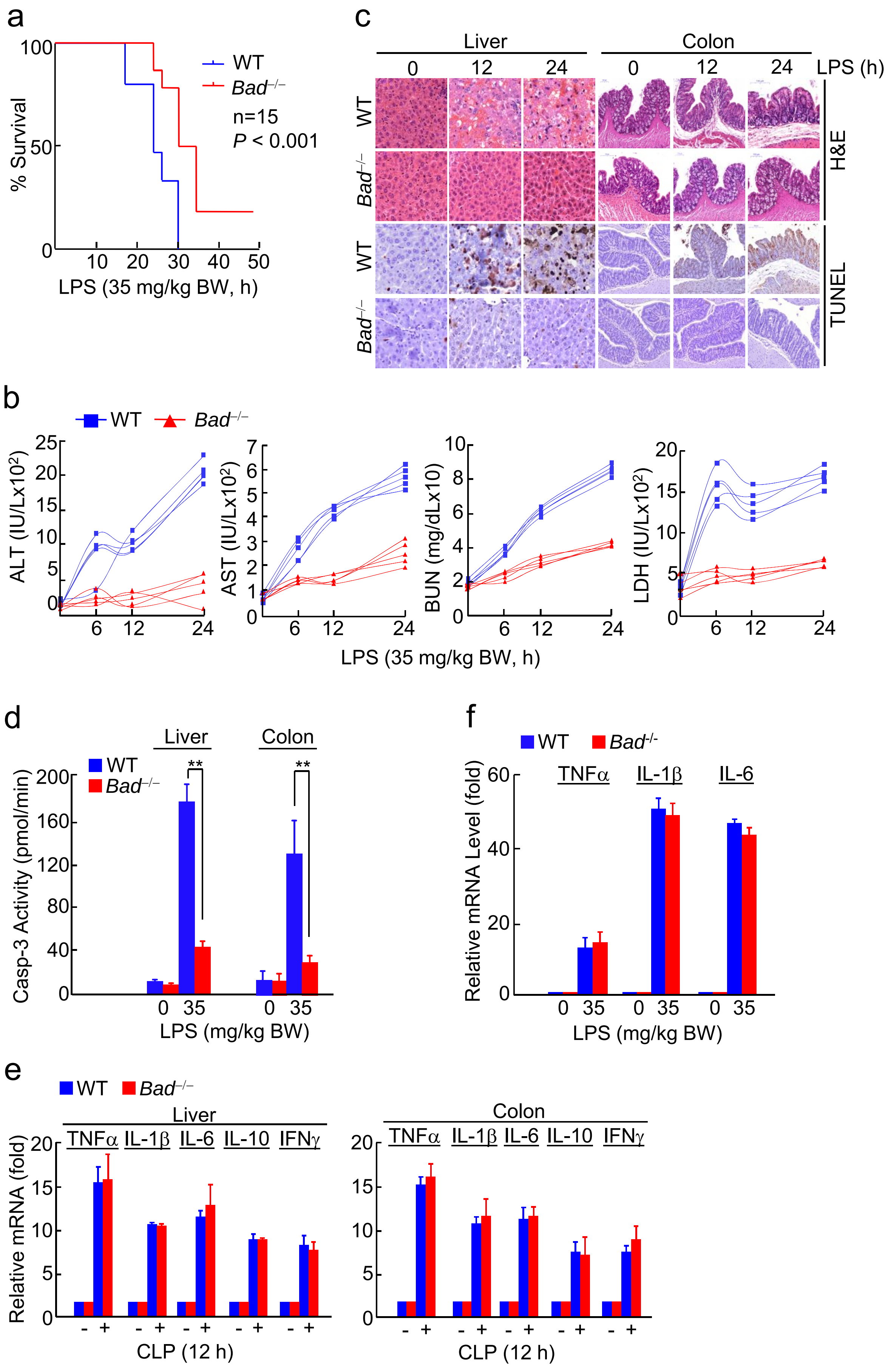
a
b
c
d
e
f
WT
Bad−/−
n=15
P < 0.001
% Survival
LPS (35 mg/kg BW, h)
Liver
Colon
LPS (h)
H&E
TUNEL
ALT (IU/Lx10²)
AST (IU/Lx10²)
BUN (mg/dLx10)
LDH (IU/Lx10²)
Casp-3 Activity (pmol/min)
LPS (mg/kg BW)
Relative mRNA Level (fold)
TNFα
IL-1β
IL-6
IL-10
IFNγ
Relative mRNA (fold)
CLP (12 h)

Supplementary Information, Figure S6 Loss of Bad protects mice from septic shock-induced tissue damage of multiorgans and mortality. (a) WT littermates and *Bad*$^{-/-}$ mice were intraperitoneally (i.p.) injected with lethal dose LPS (35 mg/kg body weight) and the mortality rate was determined. $P < 0.001$; n = 15, as analyzed by log rank (Mantel-Cox) test. **(b-d)** WT littermates and *Bad*$^{-/-}$ mice were intraperitoneally (i.p.) injected with lethal dose LPS (35 mg/kg body weight), respectively. Blood samples from the tail vein were collected at various times as indicated and the serum was used for ALT, AST, BUN and LDH determination **(b)**. Liver and colon tissues from pre-removed or at indicated times after injections were analyzed by H&E staining and TUNEL staining **(c)**. Relative Caspase 3 activity in liver and colon samples were analyzed . **, $P < 0.01$, as determined by two-tailed unpaired Student's *t* test **(d)**. **(e)** WT littermates and *Bad*$^{-/-}$ mice were subjected to high-grade CLP surgery. Expressions of TNFα, IL-1β, IL-6, IL-10 and IFNγ in liver and colon were quantitated by real-time PCR (see "Materials and Methods" for details). **(f)** *Bad*$^{-/-}$ mice and WT littermate intraperitoneally (i.p.) injected with lethal dose LPS (35 mg/kg body weight). Expressions of TNFα, IL-1β and IL-6 in liver were quantitated by real-time PCR. Data are means ±s.d. and represent three individual experiments. All results represent three individual experiments with similar results.

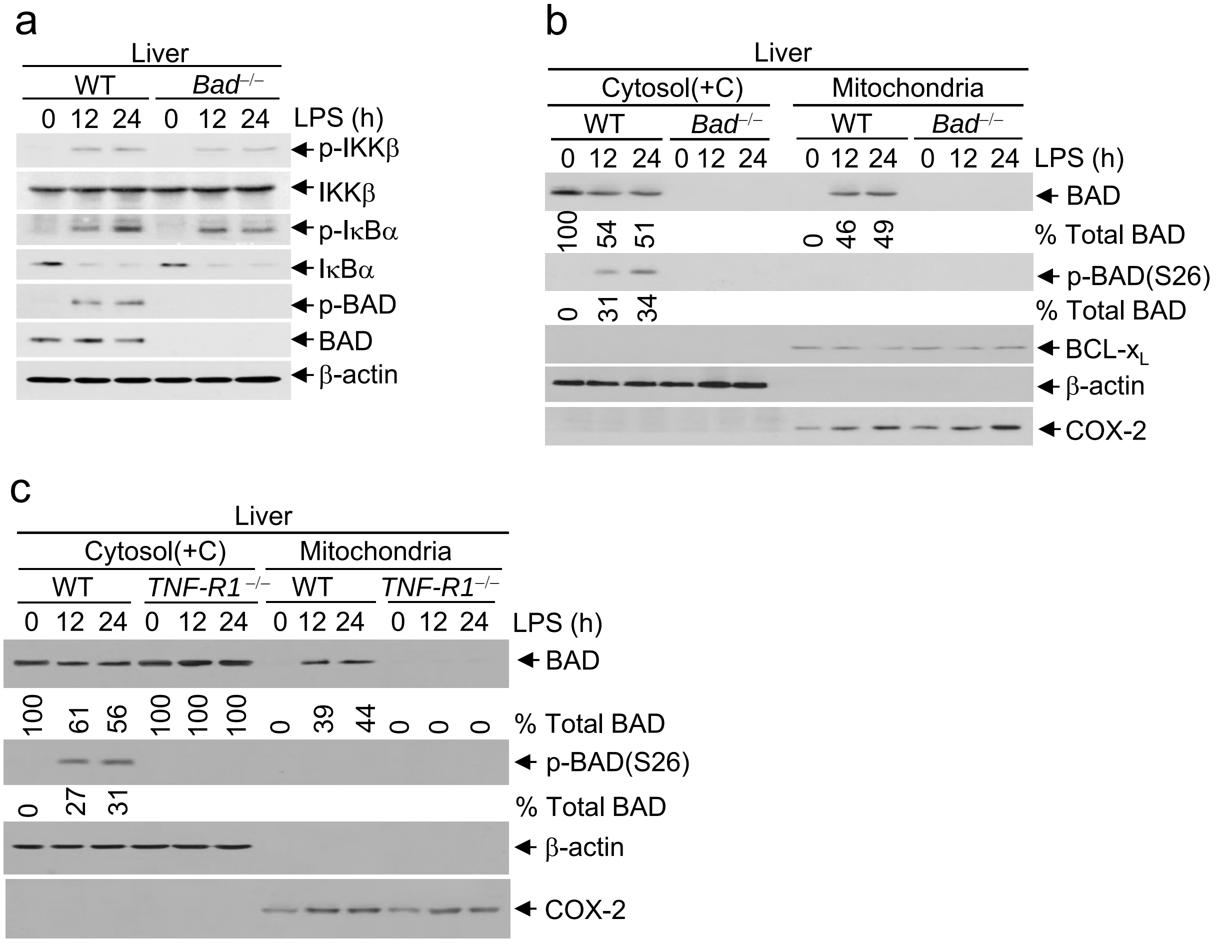

Supplementary Information, Figure S7 LPS utilizes the same mechanism as cytotoxic dose TNFα to stimulate BAD pro-apoptotic activity. *Bad*$^{-/-}$ mice and WT littermate were i.p injected with lethal dose LPS (35 mg/kg body weight). Liver tissues were extracted at different time as indicated to either analyze IKK and NF-κB activation **(a)**, or further separated into mitochondrial and cytosol [contain cytoskeleton, Cytosol(+C)] fractions to analyze BAD phosphorylation and mitochondrial translocation (see "Materials and Methods" for details) **(b)** (**Supplementary information, Figure S9z**). **(c)** *Tnf*-R1$^{-/-}$ mice and WT littermate were i.p injected with lethal dose LPS (35 mg/kg body weight). Liver tissues were extracted at different time as indicated and separated into the mitochondrial and cytosol [contain cytoskeleton, Cytosol(+C)] fractions (see "Materials and Methods" for details) to analyze BAD phosphorylation and mitochondrial translocation **(Supplementary information, Figure S9aa)**. All results represent three individual experiments with similar results.

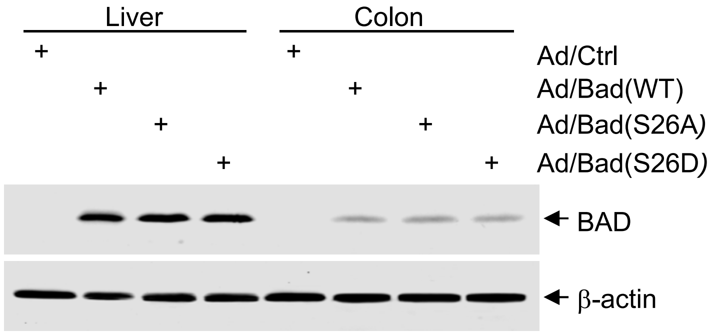

Supplementary Information, Figure S8 Expression of adenoviral vector encoding various BAD proteins (WT, S26A or S26D mutant). *Bad*$^{-/-}$ mice were intravenously injected with Ad/*WT*, Ad/*S26A*, Ad/*S26D* BAD, or Ad/*Ctrl*(LacZ) respectively for 48 h. Expression level of various BAD proteins in liver and colon were detected by immunoblotting using anti-BAD antibody.

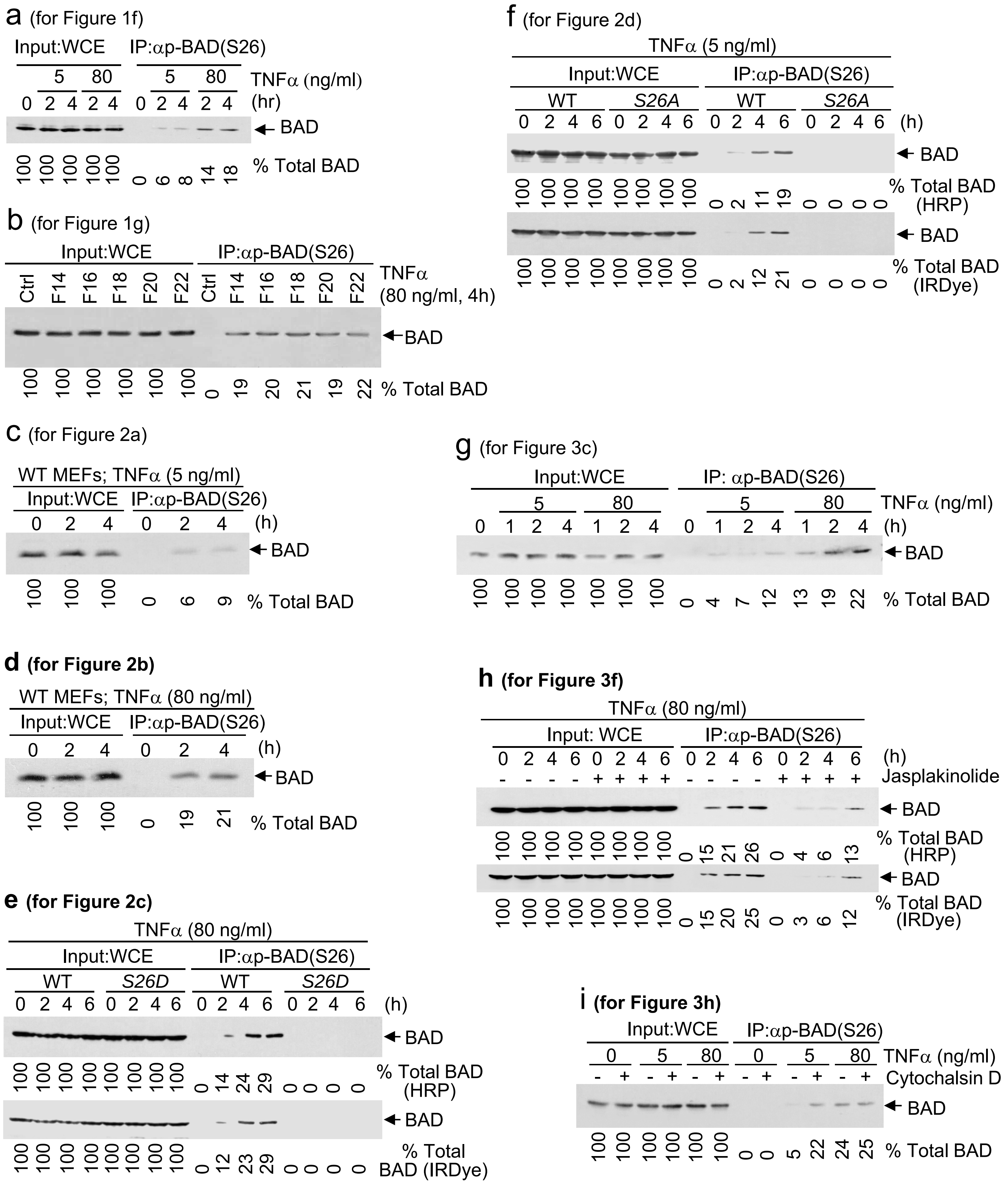
a (for Figure 1f)
Input:WCE
IP:αp-BAD(S26)
5 80 5 80 TNFα (ng/ml)
0 2 4 2 4 0 2 4 2 4 (hr)
BAD
100 100 100 100 100 0 6 8 14 18 % Total BAD
b (for Figure 1g)
Input:WCE
IP:αp-BAD(S26)
Ctrl F14 F16 F18 F20 F22 Ctrl F14 F16 F18 F20 F22
TNFα (80 ng/ml, 4h)
BAD
100 100 100 100 100 100 0 19 20 21 19 22 % Total BAD
c (for Figure 2a)
WT MEFs; TNFα (5 ng/ml)
Input:WCE IP:αp-BAD(S26)
0 2 4 0 2 4 (h)
BAD
100 100 100 0 6 9 % Total BAD
d (for Figure 2b)
WT MEFs; TNFα (80 ng/ml)
Input:WCE IP:αp-BAD(S26)
0 2 4 0 2 4 (h)
BAD
100 100 100 0 19 21 % Total BAD
e (for Figure 2c)
TNFα (80 ng/ml)
Input:WCE
IP:αp-BAD(S26)
WT S26D WT S26D
0 2 4 6 0 2 4 6 0 2 4 6 0 2 4 6 (h)
BAD
100 100 100 100 100 100 100 100 0 14 24 29 0 0 0 0 % Total BAD (HRP)
BAD
100 100 100 100 100 100 100 100 0 12 23 29 0 0 0 0 % Total BAD (IRDye)
f (for Figure 2d)
TNFα (5 ng/ml)
Input:WCE
IP:αp-BAD(S26)
WT S26A WT S26A
0 2 4 6 0 2 4 6 0 2 4 6 0 2 4 6 (h)
BAD
100 100 100 100 100 100 100 100 0 2 11 19 0 0 0 0 % Total BAD (HRP)
BAD
100 100 100 100 100 100 100 100 0 2 12 21 0 0 0 0 % Total BAD (IRDye)
g (for Figure 3c)
Input:WCE
IP: αp-BAD(S26)
5 80 5 80 TNFα (ng/ml)
0 1 2 4 1 2 4 0 1 2 4 1 2 4 (h)
BAD
100 100 100 100 100 100 100 0 4 7 12 13 19 22 % Total BAD
h (for Figure 3f)
TNFα (80 ng/ml)
Input: WCE
IP:αp-BAD(S26)
0 2 4 6 0 2 4 6 0 2 4 6 0 2 4 6 (h)
- - - - + + + + - - - - + + + + Jasplakinolide
BAD
100 100 100 100 100 100 100 100 0 15 21 26 0 4 6 13 % Total BAD (HRP)
BAD
100 100 100 100 100 100 100 100 0 15 20 25 0 3 6 12 % Total BAD (IRDye)
i (for Figure 3h)
Input:WCE
IP:αp-BAD(S26)
0 5 80 0 5 80 TNFα (ng/ml)
- + - + - + - + - + - + Cytochalsin D
BAD
100 100 100 100 100 100 0 0 5 22 24 25 % Total BAD

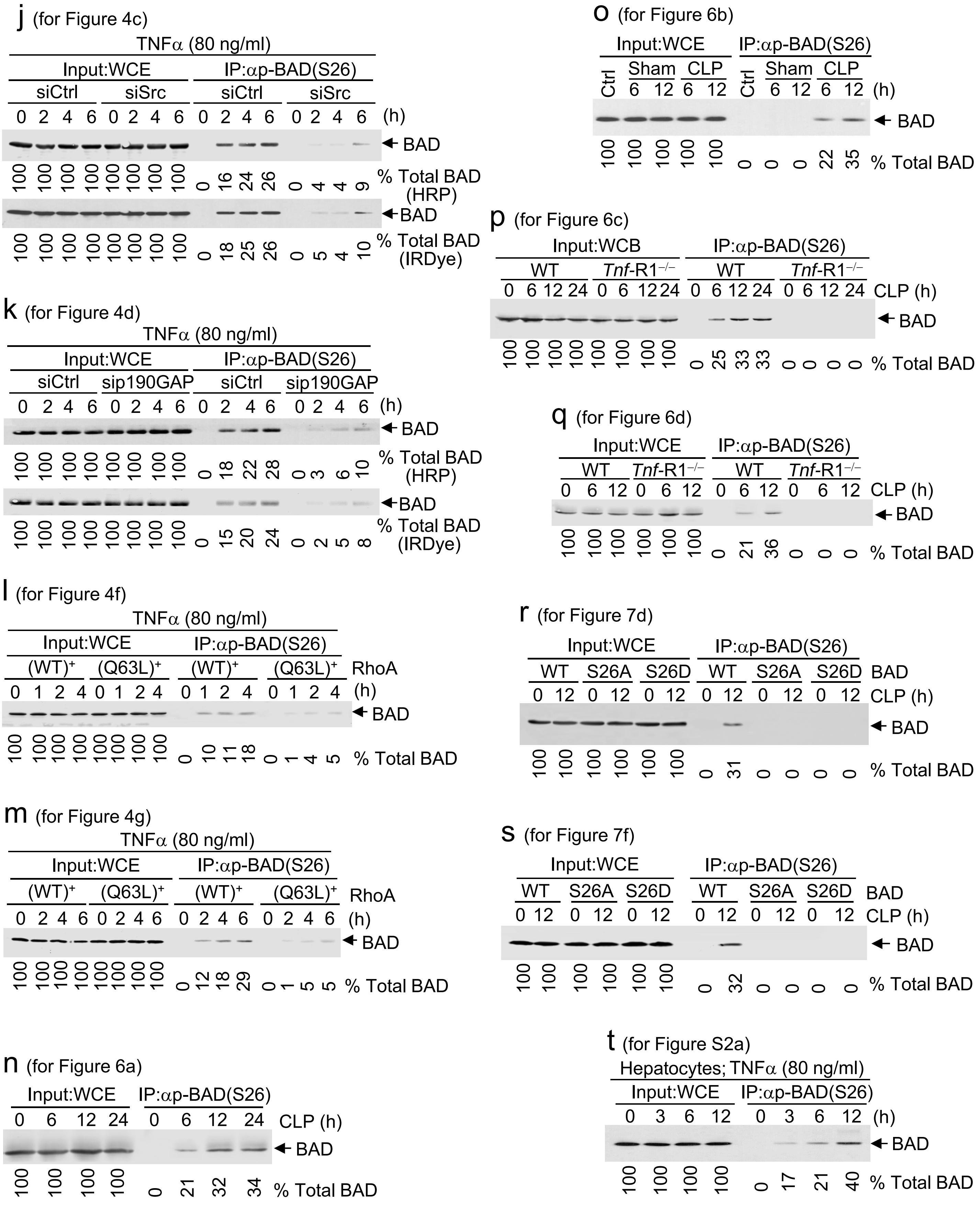
j (for Figure 4c)
TNFα (80 ng/ml)
Input:WCE
IP:αp-BAD(S26)
siCtrl siSrc siCtrl siSrc
0 2 4 6 0 2 4 6 0 2 4 6 0 2 4 6 (h)
BAD
100 100 100 100 100 100 100 100 0 16 24 26 0 4 4 9 % Total BAD (HRP)
BAD
100 100 100 100 100 100 100 100 0 18 25 26 0 5 4 10 % Total BAD (IRDye)
k (for Figure 4d)
TNFα (80 ng/ml)
Input:WCE
IP:αp-BAD(S26)
siCtrl sip190GAP siCtrl sip190GAP
0 2 4 6 0 2 4 6 0 2 4 6 0 2 4 6 (h)
BAD
100 100 100 100 100 100 100 100 0 18 22 28 0 3 6 10 % Total BAD (HRP)
BAD
100 100 100 100 100 100 100 100 0 15 20 24 0 2 5 8 % Total BAD (IRDye)
l (for Figure 4f)
TNFα (80 ng/ml)
Input:WCE
IP:αp-BAD(S26)
(WT)+ (Q63L)+ (WT)+ (Q63L)+ RhoA
0 1 2 4 0 1 2 4 0 1 2 4 0 1 2 4 (h)
BAD
100 100 100 100 100 100 100 100 0 10 11 18 0 1 4 5 % Total BAD
m (for Figure 4g)
TNFα (80 ng/ml)
Input:WCE
IP:αp-BAD(S26)
(WT)+ (Q63L)+ (WT)+ (Q63L)+ RhoA
0 2 4 6 0 2 4 6 0 2 4 6 0 2 4 6 (h)
BAD
100 100 100 100 100 100 100 100 0 12 18 29 0 1 5 5 % Total BAD
n (for Figure 6a)
Input:WCE
IP:αp-BAD(S26)
0 6 12 24 0 6 12 24 CLP (h)
BAD
100 100 100 100 0 21 32 34 % Total BAD
o (for Figure 6b)
Input:WCE
IP:αp-BAD(S26)
Ctrl Sham CLP Ctrl Sham CLP
6 12 6 12 6 12 6 12 (h)
BAD
100 100 100 100 100 0 0 0 22 35 % Total BAD
p (for Figure 6c)
Input:WCB
IP:αp-BAD(S26)
WT Tnf-R1−/− WT Tnf-R1−/−
0 6 12 24 0 6 12 24 0 6 12 24 0 6 12 24 CLP (h)
BAD
100 100 100 100 100 100 100 100 0 25 33 33 0 0 0 0 % Total BAD
q (for Figure 6d)
Input:WCE
IP:αp-BAD(S26)
WT Tnf-R1−/− WT Tnf-R1−/−
0 6 12 0 6 12 0 6 12 0 6 12 CLP (h)
BAD
100 100 100 100 100 100 0 21 36 0 0 0 % Total BAD
r (for Figure 7d)
Input:WCE
IP:αp-BAD(S26)
WT S26A S26D WT S26A S26D BAD
0 12 0 12 0 12 0 12 0 12 0 12 CLP (h)
BAD
100 100 100 100 100 100 0 31 0 0 0 0 % Total BAD
s (for Figure 7f)
Input:WCE
IP:αp-BAD(S26)
WT S26A S26D WT S26A S26D BAD
0 12 0 12 0 12 0 12 0 12 0 12 CLP (h)
BAD
100 100 100 100 100 100 0 32 0 0 0 0 % Total BAD
t (for Figure S2a)
Hepatocytes; TNFα (80 ng/ml)
Input:WCE
IP:αp-BAD(S26)
0 3 6 12 0 3 6 12 (h)
BAD
100 100 100 100 0 17 21 40 % Total BAD

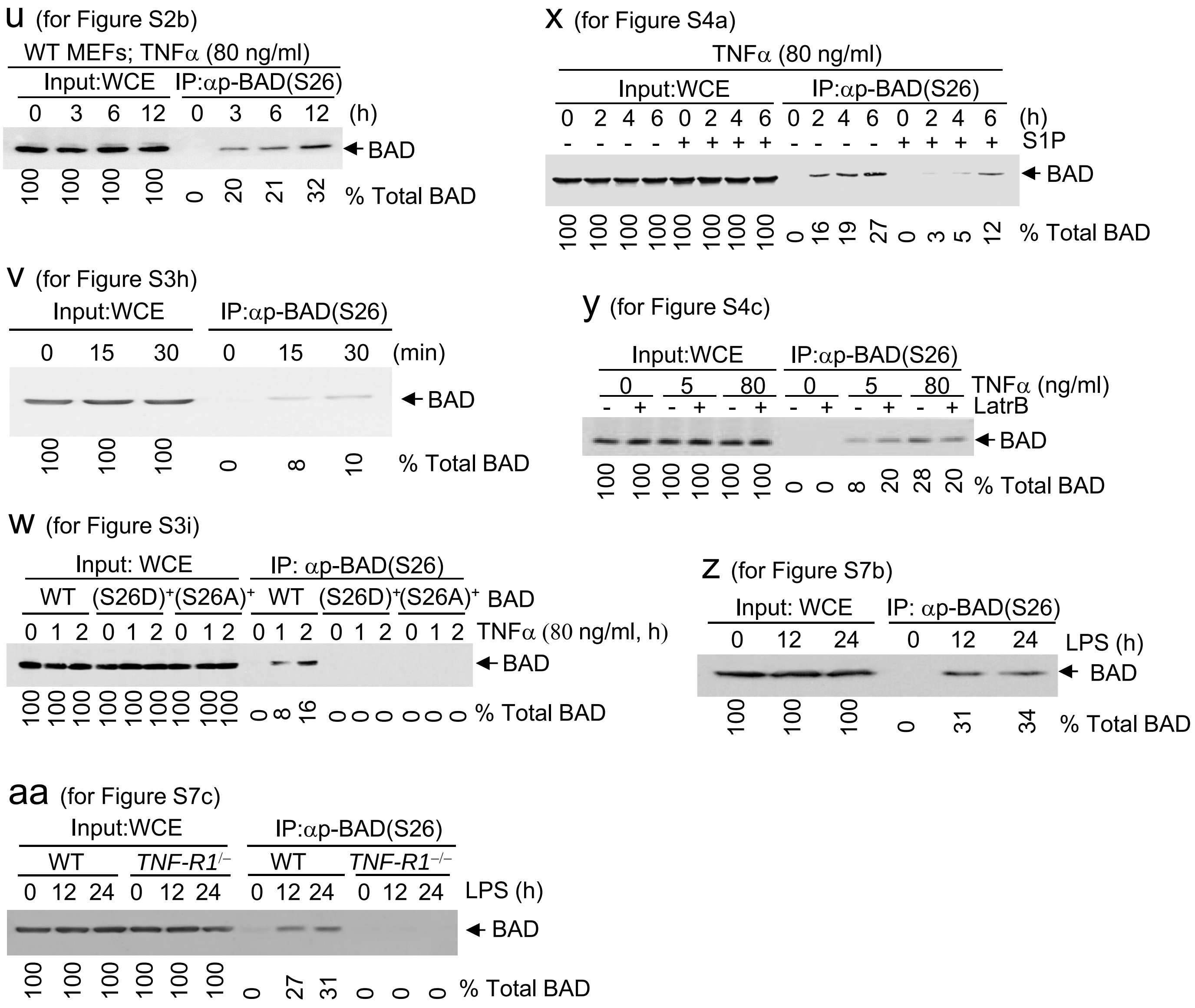

Supplementary Information, Figure S9 Quantitation of phosphorylated BAD proteins.
(a-aa) Cells were treated the same way as indicated in their corresponding figures. The percentage of IKK-phosphorylated BAD in total cytoplasmic BAD was determined by immunoprecipitation of Ser26-phosphorylated BAD with anti-Ser26 antibody in combination with immunoblotting with anti-BAD antibody, and quantitated by the ImageJ program and/or IRDye fluorescence analyzed by Odyssey Imager. BAD in the total cell extracts were calculated as 100%.

Supplementary Information, Table S1. MS/MS analysis of the TNFα preparation

Matched peptides shown in red. TNFα protein sequence coverage: 55%

1	MSTESMIRDV	ELAEEALPQK	MGGFQNSRRC	LCLSLFSFLL	VAGATTLFCL
51	LNFGVIGPQR	DEKFPNGLPL	ISSMAQTLTL	RSSSQNSSDK	PVAHVVANHQ
101	VEEQLEWLSQ	RANALLANGM	DLKDNQLVVP	ADGLYLVYSQ	VLFKGQGCPD
151	YVLLTHTVSR	FAISYQEKVN	LLSAVKSPCP	KDTPEGAELK	PWYEPIYLGG
201	VFQLEKGDQL	SAEVNLPKYL	DFAESGQVYF	GVIAL	

Start		End	Observed	Mr(expt)	Mr(calc)	ppm	score	Peptide
82	–	111	848.1648	3388.63	3388.6396	-2.8	48	R.SSSQNSSDKPVAHVVANHQV EEQLEWLSQR.A
82	–	111	678.7339	3388.633	3388.6396	-1.91	12	R.SSSQNSSDKPVAHVVANHQV EEQLEWLSQR.A
82	–	111	848.1667	3388.638	3388.6396	- 0.56	51	R.SSSQNSSDKPVAHVVANHQV EEQLEWLSQR.A
82	–	111	1130.885	3389.633	3389.6236	2.74	55	R.SSSQNSSDKPVAHVVANHQV EEQLEWLSQR.A + Deamidated (NQ)
82	–	111	848.6676	3390.641	3390.6076	9.94	47	R.SSSQNSSDKPVAHVVANHQV EEQLEWLSQR.A + 2 Deamidated (NQ)
112	–	123	615.8267	1229.639	1229.6438	-4	49	R.ANALLANGMDLK.D
124	–	144	596.075	2380.271	2380.2679	1.3	33	DNQLVVPADGLYLVYSQVLFK
145	–	160	601.6303	1801.869	1801.8781	-4.9	40	GQGCPDYVLLTHTVSR
169	–	181	471.9361	1412.787	1412.7697	11.9	33	K.VNLLSAVKSPCPK.D + Deamidated (NQ)
182	–	206	960.4846	2878.432	2878.4429	- 3.79	4	K.DTPEGAELKPWYEPIYLGG VFQLEK.G
207	–	218	635.8337	1269.653	1269.6565	- 2.85	38	K.GDQLSAEVNLPK.Y
207	–	218	636.3326	1270.651	1270.6405	8.01	31	K.GDQLSAEVNLPK.Y + Deamidated (NQ)

Supplementary Information, Table S2. IT-TOP accurate mass report of small chemicals in the TNFα preparation

#:1 Ret.Time:Averaged 1.723-49.987(Scan#:70-5561) BG
Mode:None
Mass Peaks:122 Base Peak:214.09(106631) MS Stage:MS Polarity:Pos Segment1 - Event1 Precursor:----- Cutoff: Ionization Mode:ESI

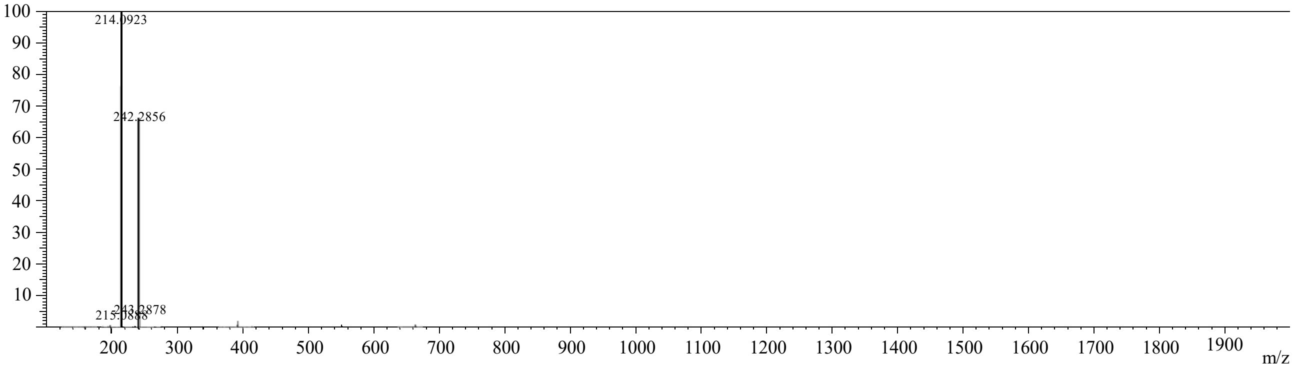

#:2 Ret.Time:Averaged 1.723-1.723(Scan#:71-71) BG Mode:None
Mass Peaks:2 Base Peak:141.00(117529) MS Stage:MS/MS Polarity:Pos Segment1 - Event2 Precursor:214.09 Cutoff:59 Ionization Model:ESI

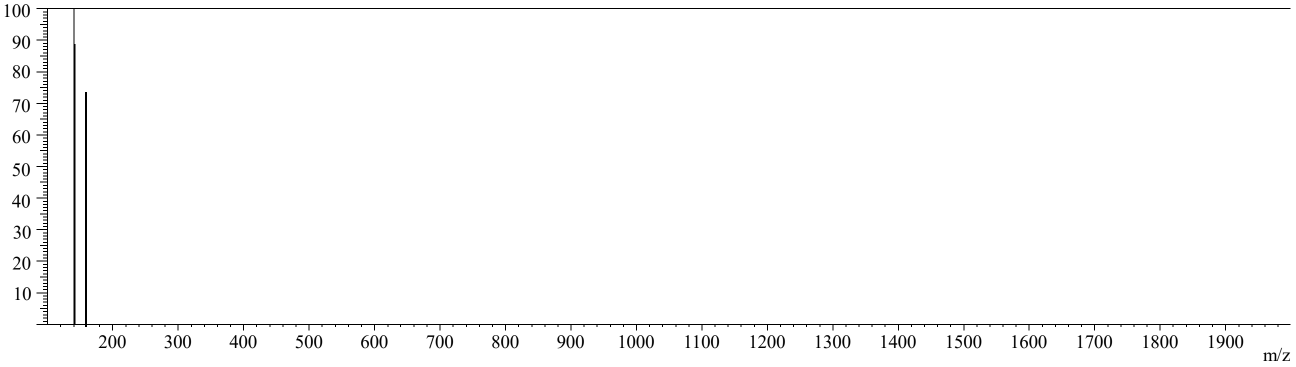

#:3 Ret.Time:Averaged 1.723-49.987(Scan#:72-5562) BG Mode:None
Mass Peaks:94 Base Peak:226.98(60912) MS Stage:MS Polarity:Neg Segment1 - Event3 Precursor:----- Cutoff: Ionization Mode:ESI

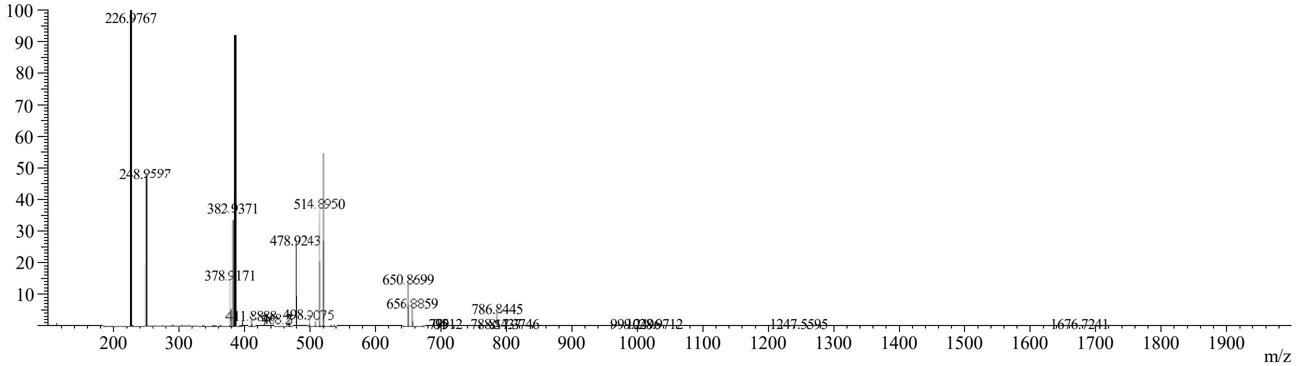

#:4 Ret.Time:Averaged 1.750-1.750(Scan#:75-75) BG Mode:None
Mass Peaks:3 Base Peak:194.96(47488) MS Stage:MS/MS Polarity:Neg Segment1 - Event4 Precursor:382.94 Cutoff:105 Ionization Mode:ESI

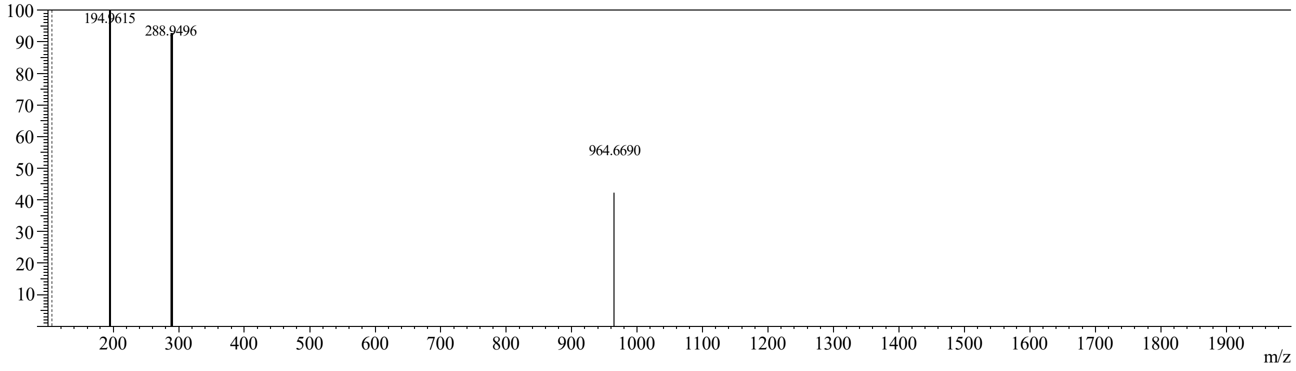

Supplementary Information, Table S3. Concentration of different fractions of the TNFα preparation

TNF fraction	PLN	Concentration (mg/mL)	Volume (mL)	Volume (mL) w/ 1% OF	Mass (mg)	100 mg/mL BSA to Add (mL)	10 mg/mL BSA to Add (mL)	Total Volume (mL)	Final Concentration (mg/mL)
9	1478329	0.0700	0.3000	0.3030	0.0210	0.0105	N/A	0.3135	0.0670
10	1478330	0.2420	0.3000	0.3030	0.0726	0.0363	N/A	0.3393	0.2140
11	1478331	0.2180	0.3000	0.3030	0.0654	0.0327	N/A	0.3357	0.1948
12	1478332	0.1980	0.3000	0.3030	0.0594	0.0297	N/A	0.3327	0.1785
13	1478333	0.1770	0.3000	0.3030	0.0531	0.0266	N/A	0.3296	0.1611
14	1478334	0.1830	0.3000	0.3030	0.0549	0.0275	N/A	0.3305	0.1661
15	1478335	0.2060	0.3000	0.3030	0.0618	0.0309	N/A	0.3339	0.1851
16	1478336	0.2520	0.3000	0.3030	0.0756	0.0378	N/A	0.3408	0.2218
17	1478337	0.2810	0.3000	0.3030	0.0843	0.0422	N/A	0.3452	0.2442
18	1478338	0.2960	0.3000	0.3030	0.0888	0.0444	N/A	0.3474	0.2556
19	1478339	0.2810	0.3000	0.3030	0.0843	0.0422	N/A	0.3452	0.2442
20	1478340	0.2510	0.3000	0.3030	0.0753	0.0377	N/A	0.3407	0.2210
21	1478341	0.2060	0.3000	0.3030	0.0618	0.0309	N/A	0.3339	0.1851
22	1478342	0.1600	0.3000	0.3030	0.0480	0.0240	N/A	0.3270	0.1468
23	1478343	0.1300	0.3000	0.3030	0.0390	0.0195	N/A	0.3225	0.1209
24	1478344	0.0960	0.3000	0.3030	0.0288	0.0144	N/A	0.3174	0.0907
25	1478346	0.0620	0.3000	0.3030	0.0186	0.0093	0.0930	0.3960	0.0470
26	1478347	0.0410	0.3000	0.3030	0.0123	0.0062	0.0615	0.3645	0.0337
27	1478348	0.0250	0.3000	0.3030	0.0075	0.0038	0.0375	0.3405	0.0220
28	1478349	0.0170	0.3000	0.3030	0.0051	0.0026	0.0255	0.3285	0.0155
29	1478350	0.0130	0.3000	0.3030	0.0039	0.0020	0.0195	0.3225	0.0121

Supplementary Information, Table S4. Correlation between the dose and biological activities of TNFα from different commercial sources (based on the manufacturer's information)

Brand	Ctrl	Low Dose	High Dose	Extra High Dose
R&D	0 [0]	5 [100-650]	80 [1,600-10,400]	
GeneScript	0 [0]	5 [>100]	80 [>1,600]	520 [>10,400]
PeproTech	0 [0]	5 [>50]	80 [>800]	1040 [>10,400]

Dose (ng/ml) [Total biological activity (Units/ml)]

Supplementary Information, Movie S1&S2

WT MEFs were treated with or without non-cytotoxic (5 ng/ml) or cytotoxic dose TNFα (80 ng/ml) for various durations, as indicated, and then analyzed by immunofluorescence staining with anti-BAD antibody for BAD protein, phalloidin for actin filament, and DAPI for nuclei, and visualized under confocal microscope with 3D program.
